# Water use efficiency of China’s terrestrial ecosystems and responses to drought

**DOI:** 10.1038/srep13799

**Published:** 2015-09-08

**Authors:** Yibo Liu, Jingfeng Xiao, Weimin Ju, Yanlian Zhou, Shaoqiang Wang, Xiaocui Wu

**Affiliations:** 1 Jiangsu Key Laboratory of Agricultural Meteorology, School of Applied Meteorology, Nanjing University of Information Science and Technology, Nanjing, 210044, China; 2 Jiangsu Provincial Key Laboratory of Geographic Information Science and Technology, Nanjing University, Nanjing, 210023, China; 3 International Institute for Earth System Sciences, Nanjing University, Nanjing, 210023, China; 4 Earth Systems Research Center, Institute for the Study of Earth, Oceans, and Space, University of New Hampshire, Durham, NH 03824, USA; 5 International Center for Ecology, Meteorology, and Environment, School of Applied Meteorology, Nanjing University of Information Science and Technology, Nanjing, 210044, China; 6 School of Geographic and Oceanographic Sciences, Nanjing University, Nanjing, 210023, China; 7 Key Laboratory of Ecosystem Network Observation and Modeling, Institute of Geographic Sciences and Natural Resources Research, Chinese Academy of Sciences, Beijing, 100101, China

## Abstract

Water use efficiency (WUE) measures the trade-off between carbon gain and water loss of terrestrial ecosystems, and better understanding its dynamics and controlling factors is essential for predicting ecosystem responses to climate change. We assessed the magnitude, spatial patterns, and trends of WUE of China’s terrestrial ecosystems and its responses to drought using a process-based ecosystem model. During the period from 2000 to 2011, the national average annual WUE (net primary productivity (NPP)/evapotranspiration (ET)) of China was 0.79 g C kg^−1^ H_2_O. Annual WUE decreased in the southern regions because of the decrease in NPP and the increase in ET and increased in most northern regions mainly because of the increase in NPP. Droughts usually increased annual WUE in Northeast China and central Inner Mongolia but decreased annual WUE in central China. “Turning-points” were observed for southern China where moderate and extreme droughts reduced annual WUE and severe drought slightly increased annual WUE. The cumulative lagged effect of drought on monthly WUE varied by region. Our findings have implications for ecosystem management and climate policy making. WUE is expected to continue to change under future climate change particularly as drought is projected to increase in both frequency and severity.

Climate change has been exerting profound influences on ecosystem function and processes^1^,^2^ including tightly coupled carbon and water cycles. Water use efficiency (WUE), defined as the amount of carbon fixed per unit water transpired^3^, measures the trade-off between carbon gain and water loss of terrestrial ecosystems. Quantifying the spatiotemporal variations of WUE and its environmental controls is of great significance to understanding the responses of terrestrial ecosystems to climate change^4^,^5^,^6^.

Ecosystem WUE is influenced by both biotic factors and abiotic factors such as leaf area index (LAI), vegetation types, radiation, temperature and precipitation^7^. Precipitation is one of the main factors that affect the spatial and temporal variations of WUE^4^,^8^. Changes of precipitation alter WUE by directly affecting ecosystem transpiration and evaporation and indirectly affecting plant carbon uptake through soil water content regulation^9^.

Both *in-situ* measurements and modeling approaches have been widely used to examine WUE. The eddy covariance (EC) technique provides continuous measurements of ecosystem-level carbon and water exchanges^10^ and has been used to assess the variations in WUE and its relationship with environment factors at different time scales (e.g. daily, monthly, and annual)^2^,^11^,^12^. Despite the growing number of EC sites, the towers have limited footprint and the resulting findings are mainly site-level understanding of WUE. It remains a challenge to scale up WUE from sites to large regions^13^. Recently, process-based ecosystem models and remote sensing approaches have been used to improve the understanding of complex carbon-water interactions and WUE functioning under changing environments at regional to continental scales^13^,^14^,^15^.

Drought is projected to become more severe and more frequent in the northern middle and high latitudes during the remainder of the 21st century^16^. It is therefore important to better understand how droughts affect ecosystem WUE. It has been hypothesized that stomatal conductance could decrease to adapt to water stress and consequently WUE could increase^9^. This hypothesis has been supported by studies based on *in-situ* measurements^17^,^18^ and modeling approaches^19^,^20^. However, several studies indicated that this hypothesis might not hold under severe drought conditions. Obvious reduction of WUE occurred^9^,^21^, especially under more severe drought^9^. A two-stage relationship between WUE and precipitation has been reported in the conterminous United States^22^. Despite these studies, how regional WUE responds to drought is still not well understood.

In this study, we integrated remote sensing data with a process-based ecosystem model - Boreal Ecosystem Productivity Simulator (BEPS) to assess spatial and temporal patterns of annual WUE of China’s terrestrial ecosystems from 2000 to 2011. The objectives of our study were to: (1) characterize the magnitude and spatial patterns of WUE of China’s terrestrial ecosystems, (2) analyze the variations of WUE over different ecoclimatic zones, and (3) assess the responses of WUE to drought.

## Results

### Magnitude and spatial distribution of annual WUE

**Figure 1:**
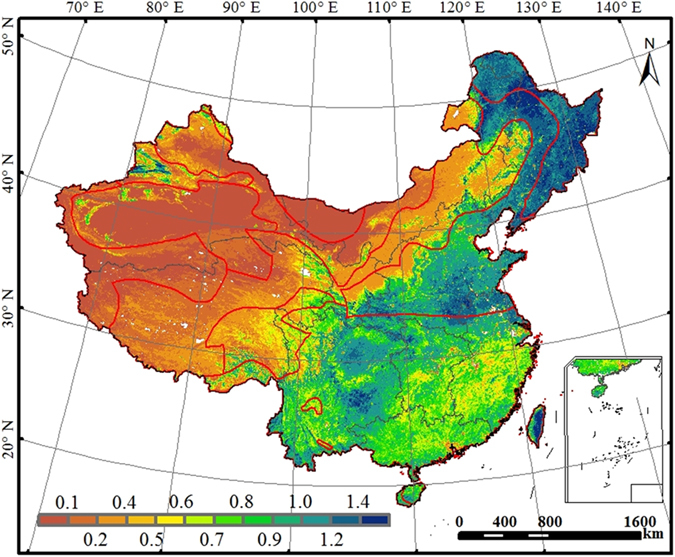
Spatial distribution of mean annual WUE (g C kg^−1^ H_2_O) over the period 2000-2011 across China. This figure was produced using ArcGIS 10.0.

The mean annual WUE of China’s terrestrial ecosystems over the period 2000-2011 showed large spatial variability owing to the influences of climate, soils, and vegetation types exhibiting large gradients across the country (Fig. 1). Deciduous forests in northeastern China had the highest annual WUE (>1.4 g C kg^−1^ H_2_O). The crop producing regions such as the Huaibei Plains, Huabei Plains, and the upper reaches of the Yangtze River also had high WUE (above 1.0 g C kg^−1^ H_2_O). Southeast China, Central China, South China, and the east of Southwest China had intermediate annual WUE ranging from 0.5 to 0.8 g C kg^−1^ H_2_O. These regions are characterized by suitable temperature and abundant precipitation and therefore have relatively high net primary productivity (NPP) and evapotranspiration (ET). The vast Tibetan Plateau, Northwest China, and the grasslands of Inner Mongolia had the lowest annual WUE (<0.4 g C kg^−1^ H_2_O) because of the low productivity and high ratio of evaporation to ET associated with sparse vegetation and low temperature.

The nationally averaged mean annual WUE simulated by BEPS was 0.79 g C kg^−1^ H_2_O. Mean annual WUE varied across regions (Fig. S1). Northeast China had the highest value (1.25 g C kg^−1^ H_2_O), followed by the cropland-dominated North China (0.97 g C kg^−1^ H_2_O). Although the annual mean NPP of Southwest China was nearly equal to that of Northeast China, higher ET in Southwest China resulted in lower annual WUE (0.93 g C kg^−1^ H_2_O). The annual WUE of Southeast China and Central China was almost identical (0.85 and 0.84 g C kg^−1^ H_2_O, respectively). The highly productive South China region had slightly lower annual WUE (0.81 g C kg^−1^ H_2_O) than Northeast China, North China, and Southwest China because of higher ET in South China. The annual WUE of Inner Mongolia (0.60 g C kg^−1^ H_2_O) was higher than that of Northwest China and Tibetan Plateau (<0.40 g C kg^−1^ H_2_O). Northwest China had the lowest annual NPP, ET, and WUE (0.34 g C kg^−1^ H_2_O) among the nine regions.

Different vegetation types differed in carbon uptake and water consumption^23^,^24^, leading to different mean annual WUE (Fig. S2). On average, deciduous broadleaf forests had the highest mean annual WUE (1.24 g C kg^−1^ H_2_O), followed by croplands (1.17 g C kg^−1^ H_2_O) and deciduous needleleaf forests (1.11 g C kg^−1^ H_2_O); evergreen forests (0.90 g C kg^−1^ H_2_O) and mixed forests (0.79 g C kg^−1^ H_2_O) had intermediate WUE; shrublands had relatively low WUE (0.69 g C kg^−1^ H_2_O); grasslands had the lowest WUE (0.39 g C kg^−1^ H_2_O).

### Trends of annual WUE

**Figure 2:**
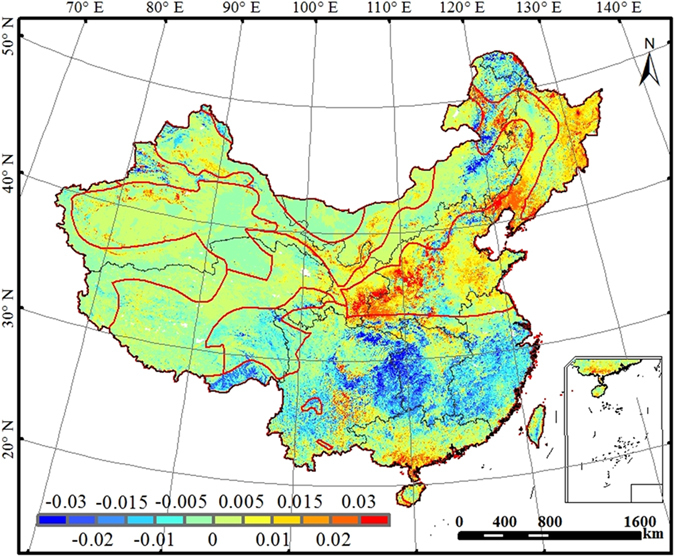
Trends of annual WUE (g C kg^−1^ H_2_O yr^−1^) for the terrestrial ecosystems across China during the period 2000-2011. This figure was produced using ArcGIS 10.0.

Annual WUE exhibited increasing trends in northern parts of China and decreasing trends in southern counterparts (Fig. 2). Annual WUE increased at a rate of 0.005 g C kg^−1^ H_2_O yr^−1^ for most regions in the eastern part of North China and a rate of 0.01 g C kg^−1^ H_2_O yr^−1^ in the eastern part of Northeast China. In the southern part of Northeast China and adjacent areas of North and Northwest China, annual WUE increased at a slightly higher rate (0.03 g C kg^−1^ H_2_O yr^−1^). South China also showed an increasing trend of WUE with a rate of >0.02 g C kg^−1^ H_2_O yr^−1^ in its southern parts. In contrast, annual WUE showed decreasing trends in northeastern Inner Mongolia, northern parts of Northeast, Tibetan Plateau, Southwest, Southeast and Central China. Southeastern Tibetan Plateau and central and southern East China exhibited decreasing trends of annual WUE (0.02 g C kg^−1^ H_2_O yr^−1^).

We also assessed the trends of spatially-averaged annual WUE for each region (Fig. S1). Although the trend of annual WUE averaged across the entire country was not statistically significant, the annual WUE showed significant decreasing trends in the southern regions: Central China (0.010 g C kg^−1^ H_2_O yr^−1^), Tibetan Plateau (0.003 g C kg^−1^ H_2_O yr^−1^), Southeast China (0.007 g C kg^−1^ H_2_O yr^−1^) and Southwest China (0.006 g C kg^−1^ H_2_O yr^−1^) and significant increasing trends in Northwest China (0.005 g C kg^−1^ H_2_O yr^−1^) (Fig. S1). Annual WUE exhibited a decreasing trend for shrublands (p < 0.05), an increasing trend for deciduous broadleaf forests (p < 0.05), and no significant trends for other vegetation types (Fig. S2).

### Vegetation effects on WUE

As one of the most important biotic factor, LAI affects vegetation growth and the partition of ET into transpiration and evaporation and exerts important controls on both NPP and ET and thereby WUE. We used the WUE-LAI space to examine the relationship between LAI and WUE over space for each vegetation type (Fig. S3). For most vegetation types, mean annual WUE first increased and then slightly declined with increasing LAI. When LAI was low, the increase in LAI could lead to the increase in both the ratio of transpiration to whole ecosystem ET and in photosynthesis and therefore result in an increase in WUE; when LAI was beyond a certain range, the variation in the ratio of transpiration to ET was constrained and WUE was therefore insensitive to the changes in LAI.

Similar change patterns of WUE with LAI have been previously reported based on EC measurement and model simulations^15^. An ecosystem-level WUE (i.e., gross primary productivity (GPP)/ET) study showed that LAI had positive influences on WUE for four grassland ecosystems in the Qinghai-Tibet Plateau and North China^25^. Tong *et al.* (2009)^26^ examined WUE of an irrigated winter wheat and summer maize rotation ecosystem in the North China Plains and found that with the increase in LAI, WUE (i.e., net ecosystem productivity (NEP)/ET) increased rapidly under low LAI but increased slowly when LAI was higher than 1.0. Kato *et al.* (2004)^27^ reported that WUE (i.e., dry matter/ET) increased with LAI when it was less than 1.6 for a sparsely planted sorghum ecosystem.

**Figure 3:**
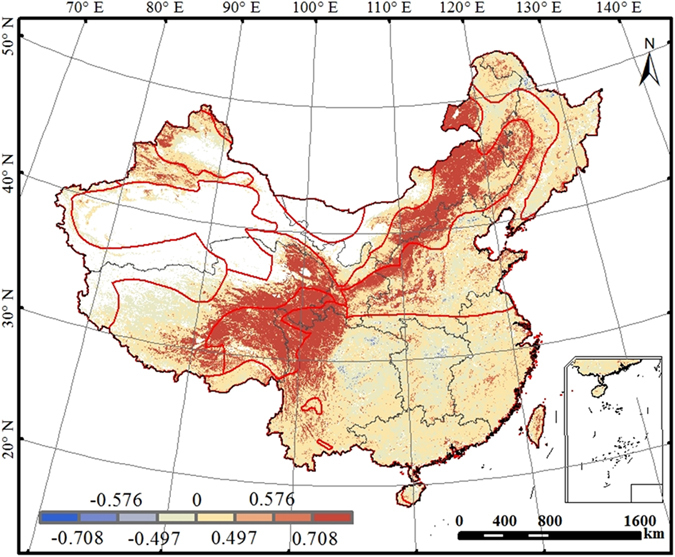
The correlation coefficients of annual mean LAI with annual WUE for terrestrial ecosystems of China during the period 2000–2011. Correlation coefficients of 0.497, 0.576, and 0.708 indicate respective significant levels of 0.10, 0.05, and 0.01. This figure was produced using ArcGIS 10.0.

LAI was partly responsible for the interannual variation of WUE. Figure 3 shows the correlation between the mean annual LAI and mean WUE during the period from 2000 to 2011. Annual LAI exhibited positive relationship with WUE in most regions. In sparsely vegetated regions, the ratio of transpiration to evaporation increases with the increase in LAI, and water is more effectively consumed for carbon uptake, leading to increases in WUE. During the 12-year period, LAI increased in northeastern and northern China^24^, and annual WUE also significantly increased (Fig. 2); LAI decreased in Qinghai-Tibet Plateau and the eastern part of Southwest China^24^, and annual WUE also significantly declined (Fig. 2).

### Drought effects on WUE

Strong relationship between WUE and precipitation had been reported^4^,^14^. The changes of simulated WUE for different vegetation types along the precipitation gradient (Fig. S4) showed that the annual WUE of evergreen needleleaf forests, evergreen broadleaf forests, deciduous needleleaf forests, mixed forests, and grasslands first increased and then decreased with annual precipitation, with the turning point varying slightly among different vegetation types (mostly around 500 mm). The effects of precipitation on annual WUE were not obvious for deciduous broadleaf forests and shrublands. In arid and semiarid regions, an increase in precipitation often induced a larger increase in NPP than in ET, leading to an increase in annual WUE. In humid and semi-humid regions, precipitation is not the major limiting factor for vegetation growth, while an increase in precipitation normally corresponds to a decrease in incoming shortwave solar radiation that is a major limiting factor for carbon uptake in the regions. In the meanwhile, increased precipitation can result in the increase of canopy interception and soil evaporation, which also contributes to the decrease in annual WUE.

**Figure 4:**
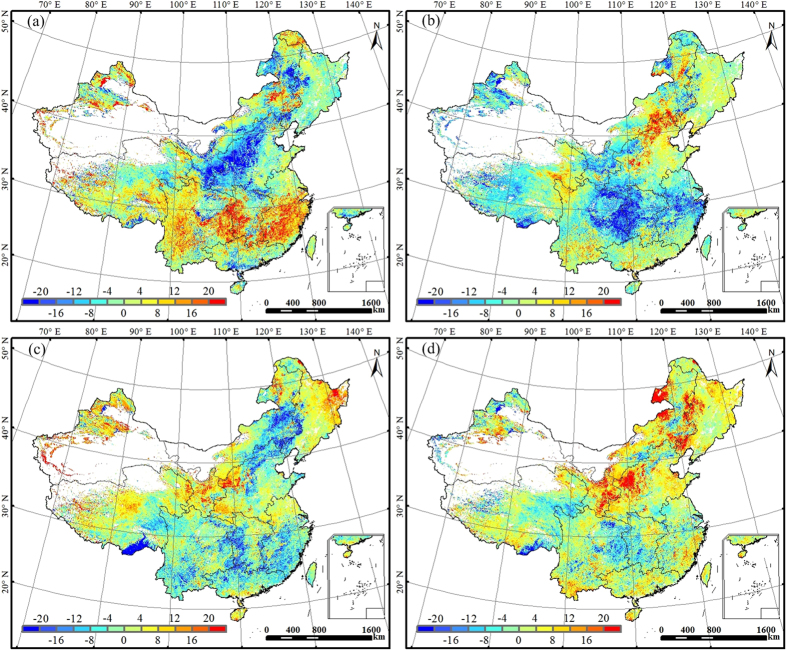
The spatial distributions of annual WUE anomalies (%) in 2001 (**a**), 2006 (**b**), 2009 (**c**) and 2011 (**d**) relative to the 12-year mean over the period 2000-2011 means, respectively. This figure was produced using ArcGIS 10.0.

Over the 12-year period from 2000 to 2011, the years of 2001, 2006, 2009, and 2011 were dry years for China^28^. WUE exhibited different responses to droughts (Figs 4 and S5). In 2001, droughts occurred in most of northern China, such as Inner Mongolia, Northeast China, and North China, and Central China^28^. Mild autumn drought in the east of Northeast China led to slight increases in annual WUE (less than 4%). Annual WUE also increased in central Inner Mongolia during moderate summer droughts. In northern Inner Mongolia suffered from mild and moderate drought in summer and autumn, however, annual WUE decreased about 16% (Fig. 4). Moderate to severe droughts in North China decreased annual WUE by more than 20%. Severe drought in the north of Northeast China did not result in significant changes in annual WUE likely because the drought led to similar reduction in annual NPP and ET (Figs 4 and S5). In this region, temperature is also a dominant controlling factor of vegetation growth, and the enhancement on NPP and ET by temperature increase during the drought period might partly offset the decrease of NPP and ET induced by water stress. Droughts hitting central China in 2006 were mostly mild^28^ and mainly occurred in summer and autumn. Annual WUE decreased by more than 16% in northern Central China, central Southeast China, and southern Tibetan Plateau. Moderate summer drought occurred in regions around Chongqing decreased annual WUE by about 8% (Fig. 4). By contrast, annual WUE increased in the north of North China affected by mild autumn drought. In mid drought-hit regions of Inner Mongolia and North China, annual WUE increased by more than 20%. In 2009, mild to moderate droughts affected a large portion of China. Inner Mongolia and Northeast China were hit by droughts in spring and summer while the south of Southwest China was affected by droughts in autumn and winter. These droughts induced a larger decrease in NPP than in ET, leading to a decrease in annual WUE by 16–20% (Fig. 4). In contrast, summer drought-induced reduction in ET was larger than the decline in NPP across the Northwest China, leading to increases in annual WUE (Figs 4 and S5). In Tibetan Plateau, mild to moderate droughts in summer and autumn increased annual WUE in the north and decreased WUE in the southeast (Fig. 4). The drought in 2011 was perhaps the most severe one over the last five decades. The southern half of the country experienced moderate to extreme droughts in spring and winter^28^. The droughts in Southwest, Central, Southeast, and South China resulted in similar reduction in NPP and ET, and therefore there were no large changes in annual WUE (Figs 4 and S5). The extreme spring and summer droughts hitting Yunnan and Jiangxi provinces caused slight reduction in annual WUE (by ∼10%). Mild and moderate autumn drought occurring in Northeast China and central Inner Mongolia increased annual WUE by ∼8% (Figs 4 and S5).

The responses of WUE to drought varied with drought severity. In the four dry years, slight droughts usually resulted in increases in annual WUE in Northeast China and central Inner Mongolia but declines in Central China (Fig. 4). In southern China, moderate and extreme drought caused WUE declines, while severe drought often resulted in slight increases. The timing of drought was also partly responsible for the different responses of WUE to drought. For example, the severe droughts occurred in spring of 2001 led to significant decreases in annual WUE in the north of North China, while the similarly severe autumn drought in 2006 only slightly increased WUE (Figs 4 and S5). That can be explained by the different responses of NPP and ET to droughts in spring and autumn in northern parts of China. Spring drought normally exerted negligible effects on NPP but increased ET, while autumn drought resulted in greater decrease in ET than in NPP, and consequently induced the different responses of annual WUE in these regions. While in southern parts of China this phenomenon was not obvious.

**Figure 5:**
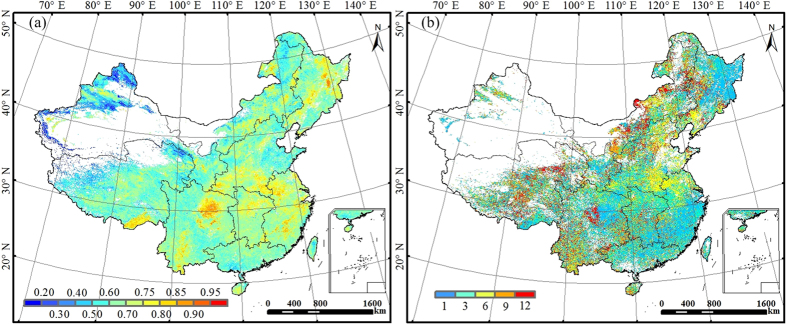
Spatial distribution of the correlations between monthly WUE and SPI for the period of 2000–2011. (**a**) The values represent the maximum correlation recorded (Pearson coefficient, R) for each pixel, independently of the month of the year and the SPI time-scale. (**b**) SPI time-scales at which the maximum correlation between SPI and WUE recorded. Areas with no significant correlations (p>0.05) are indicated in white. This figure was produced using ArcGIS 10.0.

Drought and the resulting changes in WUE may not occur simultaneously because of the different responses of carbon uptake and ET to drought. The correlation coefficient (Pearson coefficient, R) between monthly WUE values and monthly standardized precipitation index (SPI) at different time scales were calculated to assess the accumulative lagged effects of droughts on WUE. Figure 5 shows the spatial distribution of maximum correlation coefficient between SPI and monthly WUE for the period 2000–2011 and the corresponding SPI timescales. WUE was significantly correlated with SPI (p < 0.05) in more than 68% vegetated areas (Fig. 5a). SPI and WUE exhibited strongest relationship in central, southwestern, and northeastern China affected by several droughts during the study period. Weak relationships between SPI and WUE were observed only in some western regions.

The SPI timescale at which maximum correlation between SPI and WUE occurred varied over space (Fig. 5b). The identified response patterns were generally associated with vegetation types and local natural conditions. The accumulative lagged effects were relatively short (e.g., 1–3 months) in the southeastern China and were relatively long (6–9 months) in southwestern regions. In cropland-dominated regions like the east of North China, WUE responded to droughts at intermediate timescales of about 6 months. WUE responded to drought with the longest time lag (more than 9 months) in grassland-dominated areas from Inner Mongolia to the Qinghai-Tibet Plateau. Similar responses of vegetation to different time scales of droughts in different global biomes was recently reported^29^.

## Discussion

The WUE values of different vegetation types simulated by BEPS were generally consistent with the EC-derived WUE. The WUE of forests and croplands was higher than that of grasslands^13^,^17^,^30^. Croplands usually have relatively high WUE values under sufficient water supply^26^, and the WUE is close to that of evergreen needleleaf forests^31^. The averaged mean annual WUE of West China in 2002 estimated using C-FIX and CoLM model was about 0.32 g C kg^−1^ H_2_O^32^, which was lower than our estimates. The spatial patterns of averaged annual WUE of the Yangtze River Basin from 1956 to 2006 simulated by the IBIS model^33^ were similar to our simulation.

We compared our annual WUE simulated by BEPS against values derived from MODIS NPP and ET products. The spatial patterns of BEPS and MODIS-based WUE were generally consistent in most regions (Fig. S6). There were some discrepancies in northeastern, northern, and southwestern China. For example, the MODIS-based WUE (∼0.6–0.9 g C kg^−1^ H_2_O) in northeastern China was much lower than BEPS-based WUE (∼1.2–1.4 g C kg^−1^ H_2_O). Our higher WUE values in northeastern China were supported by EC flux tower measurements. The ratio of GPP/ET for deciduous forests in northeastern China was 2.57 g C kg^−1^ H_2_O^34^, and therefore the NPP/ET ratio was equal to 1.29 g C kg^−1^ H_2_O assuming that the NPP/GPP ratio is 0.5^35^,^36^. Croplands in northern China should have relatively high WUE values under sufficient water supply^26^,^31^. Both BEPS-based WUE and MODIS-based WUE exhibited similar trends at the national scale during the period 2000–2011 with decreasing trends mainly in the south and increasing trends mainly in the north (Fig. S6).

Our nationally-averaged annual WUE of China’s terrestrial ecosystems (0.79 g C kg^−1^ H_2_O) was slightly higher than the mean annual WUE of the southern United States (0.71 g C kg^−1^ H_2_O) during 1895-2007^14^, but lower than the global mean annual WUE (0.92 g C kg^−1^ H_2_O) averaged for the period from 1995 to 2004^13^. The simulated average WUE of the southern United States biomes followed an order of: forests (0.93 g C kg^−1^ H_2_O), grasslands (0.58 g C kg^−1^ H_2_O), croplands (0.54 g C kg^−1^ H_2_O), and shrublands (0.45 g C kg^−1^ H_2_O)^14^, while the global WUE showed forest ecosystems has the highest value (1.10 g C kg^−1^ H_2_O), followed by croplands (0.85 g C kg^−1^ H_2_O), grasslands (0.81 g C kg^−1^ H_2_O), dense shrublands (0.80 g C kg^−1^ H_2_O), and open shrublands (0.67 g C kg^−1^ H_2_O)^13^. In this study, simulated mean annual WUE in China was in the following order: croplands (1.17 g C kg^−1^ H_2_O) > forests (0.84 g C kg^−1^ H_2_O) > shrublands (0.69 g C kg^−1^ H_2_O) > grasslands (0.39 g C kg^−1^ H_2_O). Our WUE estimates were higher for croplands and lower for grasslands than those of the southern United States^14^ and over the globe^13^. The inconsistency of WUE for the same ecosystems in different studies can be partly attributed to the diverse climate and vegetation conditions of croplands and grasslands and to different approaches used.

Based on EC data, Yu *et al.* (2008)^34^ found that WUE (i.e., GPP/ET) of forest ecosystems decreased from south to north along the North-South Transect of Eastern China. This decreasing trend was supported by foliar δ^13^ C measurements^37^. An EC-based synthesis study showed that the WUE (i.e., GPP/ET) of terrestrial ecosystems in China increased with precipitation quickly when annual precipitation was below 500 mm, but saturated when precipitation was higher than 500 mm^11^. WUE maps derived from empirical, statistical approaches showed abrupt changes of WUE (i.e., GPP/ET) occurred along the 400–500 mm precipitation climatic isocline boundary of arid and semi-humid zones^38^. These findings were generally consistent with our results.

At the national scale, the dynamics of WUE was dominated by the variations of ET (Fig. S5). The detectable increase in national annual ET (∼0.8 mm yr^−1^) was partly responsible for the slight decrease in national mean annual WUE. The two components of WUE (NPP and ET) played different roles in controlling the variations of annual WUE over different regions. The variations in annual WUE in Northeast China, Inner Mongolia, North China, Southeast China, and South China were mainly caused by the changes in NPP, whereas the inter-annual changes in WUE in Northwest China, Central China, Tibetan Plateau, and Southwest China were mainly caused by the variability in annual ET (Fig. S5). The negative correlation between NPP and WUE in southern China was consistent with the previous finding based on EC data that the seasonal and annual variations of WUE (i.e., GPP/ET) were negatively correlated with the changes in GPP^34^. Drought led to decreasing NPP^23^ but increasing ET^24^ in many areas of southern China, resulting in decreases in WUE.

Ecosystem WUE calculated from the ratio of NPP to ET can also be expressed as: WUE = (NPP/T) * (T/ET)^30^. NPP/T is the transpiration-based WUE measuring how much dry matter was produced through plant transpiration (T), while T/ET, the partitioning of evapotranspiration into transpiration, describes how water vapor flux is allocated between ecosystem biological and physical processes^7^,^25^. The interannual dynamics of ecosystem WUE was controlled by the variations of NPP/T and T/ET. During the 12-year study period, NPP/T decreased in most southern regions of China, while T/ET increased in most regions of China (Fig. S7). The decrease in NPP/T was larger than the increase in T/ET in corresponding regions, resulting in the decrease in WUE (Fig. 2). The responses of an ecosystem to drought are associated with its adaptation and resilience to climate change. Drought usually reduces plant transpiration and total ecosystem ET. Owing to the conservative water-use strategies^25^, plants in arid regions maintain relatively high NPP/T to survive under drought, resulting in slight changes in WUE. Water is not the main limiting factor of carbon uptake in humid regions^39^, and drought accompanied with abundant solar radiation can enhance plant growth rates and lose more water through transpiration due to dense canopies^25^. A decrease in NPP/T and an increase in T/ET led to a decrease in WUE.

Our findings can help land managers and policy makers better understand ecosystem responses to climate change and implement climate-related policies. Our study found that droughts occurred more extensively and frequently in China during the past decade, especially in southern China. The increased droughts would exert significant influence on the ecosystem productivity and health. Drought-induced increase of annual WUE in Northeast China and central Inner Mongolia indicates that (1) ecosystems suffering from long-term water deficit have adaptation strategies to water stress; and (2) national policies including the “Grain-for-Green” Program have increased NPP in these regions^40^ despite water stress. WUE reflects the tradeoffs between the carbon gain and water loss of ecosystems. There are often conflicts in balancing ecosystem carbon uptake and water consumption^14^. The increasing WUE in northern regions indicates that afforestation is likely feasible in at least some of these regions despite limited water availability. The “turning-points” observed for southern China, i.e., moderate and extreme droughts reduced annual WUE, while severe drought slightly increased annual WUE suggests an optimum water management strategy in vast southern parts of China with plentiful precipitation.

Although the BEPS model has been successfully used to quantify water and carbon fluxes for different ecosystems, it should be noted that, as many other ecosystem models, there are still some errors in model simulations resulting from uncertainties in model structure, inputs, and parameterization schemes^41^,^42^. Like other ecosystem models, BEPS contains some imperfect ecosystem processes and a set of assumptions. Moreover, certain important processes have not yet been incorporated into BEPS. For example, management practices (e.g., irrigation, logging) have not been included in the model. The biases in model inputs including meteorological and LAI data could also introduce significant uncertainties to the modeled fluxes and WUE. In addition, although the BEPS model has been carefully parameterized based on EC tower site measurements, field observation data, and previous literature-reported values^23^,^24^,^43^,^44^, some key parameters may still exhibit significant uncertainties.

Six different simulations were conducted to assess the sensitivity of NPP, ET, and WUE to LAI, precipitation, and temperature (Fig. S8). In each simulation scenario, only one variable was changed by a certain magnitude for all pixels while all other inputs remained undisturbed. The national annual NPP, ET and WUE increased by 11.6%, 5.4%, and 5.9%, respectively, when LAI increased by 25%; NPP, ET, and WUE decreased by 15.1%, 6.2%, and 9.5%, respectively, when the LAI decreased by 25%. An increase in precipitation by 25% resulted in a slightly lower increase in NPP (3.0%) than in ET (5.4%), leading to a decrease in WUE (2.3%); a decrease in precipitation by 25% led to a slightly higher decrease in national ET (8.8%) than in NPP (6.3%), resulting in an increase of WUE (2.7%). The national annual WUE decreased by 7.2% and increased by 6.8% when the temperature increased and decreased by 1 °C, respectively. Our sensitivity analysis showed that NPP, ET, and WUE were sensitive to large changes in LAI, precipitation, and temperature. This indicates that the biases in model inputs can introduce uncertainties to modeled WUE. The analysis also indicates that future changes in climate change and LAI are expected to influence WUE of China’s terrestrial ecosystems.

## Conclusions

In this study, the BEPS model along with climate and remotely-sensed LAI data was used to examine the magnitude, patterns, and trends of WUE and its underlying drivers for China’s terrestrial ecosystems over the period from 2000 to 2011. The average annual WUE of China’s terrestrial ecosystems was 0.79 g C kg^−1^ H_2_O and exhibited variations among vegetation types, with the highest values for deciduous forests and croplands and the lowest value for grasslands. During the 12-year period, annual WUE exhibited decreasing trends in the southern regions because of decreases in NPP and increases in ET and increasing trends in the most northern regions mainly because of the increases in NPP. The annual WUE increased with the increase of LAI in areas with low LAI and slowly increased or even declined with the increase of LAI in areas with high LAI. The annual WUE first increased and followed by decrease with annual precipitation, with a threshold value of around 500 mm. Droughts usually resulted in increased annual WUE in Northeast China and central Inner Mongolia but declined WUE in Central China in the four dry years: 2001, 2006, 2009, and 2011. “Turning-points” were observed for southern China: moderate and extreme droughts reduced annual WUE, while severe drought slightly increased annual WUE. The cumulative lagged effect of drought on monthly WUE was 1-3 months for mostly forested southeastern regions, ∼6 months for cropland-dominated North China, and >9 months for grassland-dominated regions.

Our results have implications for ecosystem management and climate policy making. Ecosystems suffering from long-term water deficits in northern regions have adaptation strategies to water stress. The increasing WUE in these regions also indicates that afforestation is likely feasible despite water stress. “Turning-points” observed for southern China suggests an optimum water management strategy in vast southern parts of China with plentiful precipitation. WUE is expected to continue to change under future climate change particularly as drought is projected to increase in both frequency and severity.

## Methods

### Model description

The ecosystem model used here is the BEPS model, and it originally stemmed from the FOREST biogeochemical cycles (FOREST-BGC) model^45^. BEPS includes photosynthesis, hydrological, energy balance, and soil biogeochemical modules^46^. It stratifies whole canopies into sunlit and shaded leaves to calculate daily carbon uptake and transpiration for these two groups of leaves separately. Daily GPP is calculated using Farquhar’s instantaneous leaf biochemical model up-scaled with a new temporal and spatial scaling scheme^47^. The BEPS model has been continuously improved and extensively applied to simulate carbon and water fluxes in a wide variety of terrestrial ecosystems^48^. More details about this model have been fully documented elsewhere^45^,^46^,^47^,^49^.

### Model inputs

In this study, the BEPS model was driven by spatially invariant atmospheric CO_2_ concentration data, temporally invariant soil data, and spatially and temporally explicit remote sensing and meteorological data spanning the period from 2000 to 2011.

- (1) Remote sensing data: Yearly MODIS land cover datasets (MCD12Q1 V051) from 2001 to 2011^50^ and 8-day LAI from 2000 to 2011 with a spatial resolution of 500 m. The LAI data used here were inverted using the MODIS reflectance product (MOD09A1 V05), MODIS land cover datasets and a 4-scale geometric optical model based algorithm^51^. Previous studies showed that this algorithm is superior to the algorithm used to produce the MODIS LAI product^52^.
- (2) Meteorological data: Gridded meteorological data (including daily maximum and minimum air temperatures, precipitation, relative humidity, and incoming solar radiation) with a spatial resolution of 500 m. The gridded data were interpolated from observations of 753 weather stations across China using the inverse distance weight (IDW) method. A lapse rate of 0.6 °C per 100 m was used for the interpolation of temperatures. Sunshine duration measurements were used to estimate incoming solar radiation for stations without solar radiation observations^53^. The accuracy of the gridded meteorological data was assessed using the measurements at 8 EC tower sites of ChinaFLUX and monthly 0.5° grid data from China Meteorological Administration (CMA). Our gridded data generally agreed well with the EC data at the site level and the CMA data at the national scale (Figs S9-S12).
- (3) Soil data: The volumetric fractions of sand, clay, and silt were interpolated from the soil texture maps developed by Shangguan *et al.* (2012)^54^ based on the 1: 1,000,000 scale soil map of China and 8595 soil profile records in the second national soil survey dataset.

### Model parameterization and validation

Prior to the national simulation in China, the BEPS model was parameterized using tower measurements^23^,^24^ and literature values^43^,^44^. The model has been re-parameterized for its application in China. The key biophysical and biochemical parameters used for different vegetation types are listed in Table S1.

Numerous validation efforts have demonstrated that BEPS can simulate carbon and water fluxes well at a variety of spatial and temporal scales. The ability of the BEPS model to simulate GPP, NPP, and ET of China’s terrestrial ecosystems was recently evaluated against tower-based carbon and water flux data measured at ChinaFLUX sites and NPP data from forest inventory and tree ring cores^23^,^24^. The evaluation confirmed the good performance of the BEPS model in simulating GPP, NPP, and ET of different ecosystem types at daily and annual scales. Recently, modeled WUE was compared against WUE (i.e., GPP/ET) derived from measurements at 18 flux sites in East Asia, indicating that BEPS could simulate the magnitude and variations of monthly mean WUE well^15^.

**Figure 6:**
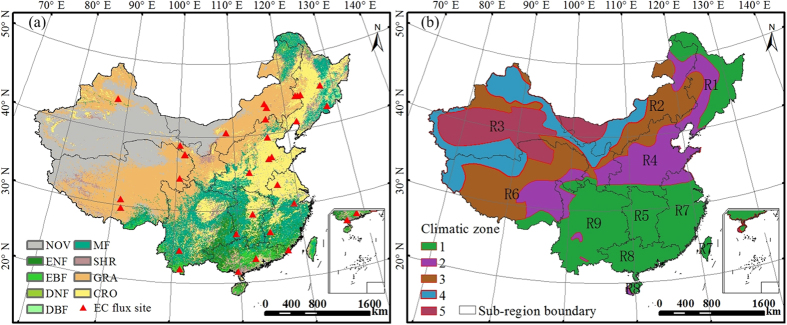
(**a**) Distribution of the eddy covariance (EC) flux sites with data used in this study. The base map is the reclassified 2011 MCD12Q1. (Land-cover types: evergreen needleleaf forests (ENF), deciduous needleleaf forests (DNF), evergreen broadleaf forests (EBF), deciduous broadleaf forests (DBF), mixed forests (MF), shrublands (SHR), croplands (CRO), grasslands (GRA), non-vegetation (NOV)). (**b**) Depiction of five climatic zones and nine sub-regions in China. (Five climatic zones: 1: humid zone, 2: sub-humid zone, 3: semiarid zone, 4: arid zone, 5: extreme arid zone. Nine sub-regions: R1: Northeast China, R2: Inner Mongolia, R3: Northwest China, R4: North China, R5: Central China, R6: Tibetan Plateau, R7: Southeast China, R8: South China, and R9: Southwest China). This figure was produced using ArcGIS 10.0.

**Figure 7:**
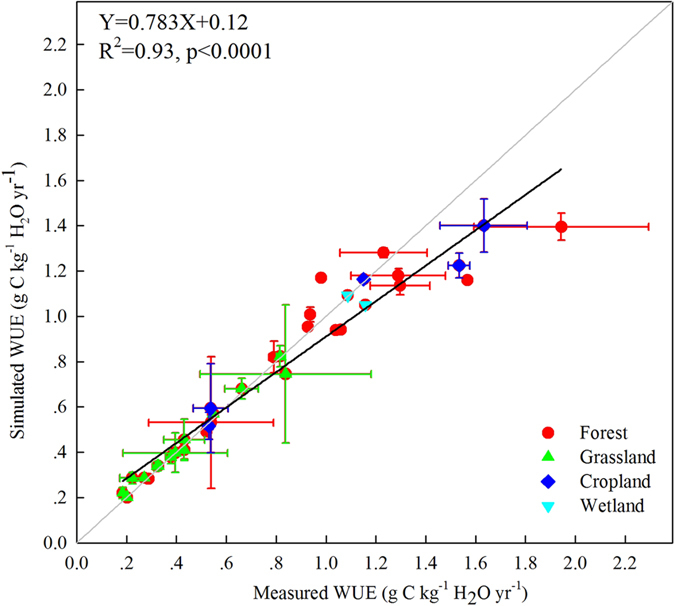
Validation of BEPS simulated annual WUE against WUE derived from EC measurements. The error bars indicate standard deviation of annual WUE for each site.

In this study, we further used EC data from 35 sites (13 forest sites, 14 grassland sites, 5 cropland sites, and 3 wetland sites)^11^,^25^,^34^,^55^ to evaluate the performance of BEPS in simulating WUE across China (Fig. 6 and Table S2). The annual MODIS NPP and ET products were also used to calculate WUE and to compare against annual WUE simulated by the BEPS model. The NEP data observed at EC sites were partitioned into GPP and ER. In order to calculate tower WUE (NPP/ET), tower GPP should be converted to NPP. As the residual of GPP minus autotrophic respiration, NPP can be treated as a fixed fraction of GPP at longer time step with uncertainties^35^,^36^. Mean values of NPP/GPP over the EC tower sites simulated by BPES are 0.43 ± 0.10 for forest, 0.48 ± 0.07 for grassland, 0.54 ± 0.06 for cropland, and 0.54 ± 0.01 for wetland, respectively. These values were consistent with the previous observation synthesis and modeling studies^35^,^36^. We converted tower GPP to NPP using the ratio of NPP to GPP simulated by BEPS for each site and then compared the tower-derived annual WUE with modeled WUE across the sites. The comparison showed that the BEPS model could simulate the annual WUE across sites fairly well (Y = 0.78X + 0.12, R^2^ = 0.93, p < 0.0001) (Fig. 7). The BEPS model slightly overestimated WUE in the lower end of the range (<0.4 g C kg^−1^ H_2_O) and slightly underestimated WUE in the higher end of the range (>0.8 g C kg^−1^ H_2_O). The ratios of NPP to GPP simulated by BEPS were used to convert tower-based GPP to NPP. To provide a more independent validation, we also validated modeled GPP/ET using EC-derived GPP/ET. The comparison showed that BEPS simulated WUE with the definition of GPP/ET fairly well for 35 EC tower sites across China (Fig. S13).

### Analysis

WUE has a variety of definitions^56^, and the ratios of different measures of carbon gain (e.g., gross primary productivity (GPP), net primary productivity (NPP), aboveground NPP (ANPP), net ecosystem productivity (NEP)) and water loss (e.g., transpiration (T), evapotranspiration (ET)) have been used to define ecosystem WUE^30^. The ratio of NPP to ET is perhaps one of the most widely used definitions for assessing WUE at regional or global scales^13^,^14^,^20^. Ecosystem WUE was defined using the ratio of NPP to ET (WUE = NPP/ET) in this study.

For each year over the period 2000–2011, we calculated annual WUE from annual NPP and ET simulated by BEPS for each grid cell. We then assessed the magnitude and spatial patterns of WUE at the national scale and across different vegetation types. To analyze spatial and temporal patterns of WUE and its responses better, five ecoclimatic zones according to secular annual mean aridity and nine sub-regions according to provincial administrative boundaries were adopted as divisions of China (Fig. 6). Annual WUE was averaged across the entire country, each vegetation type, and each sub-region to produce spatially-averaged time series.

We also assessed the trends of annual WUE for the 12-year study period using the linear fitting method (Y=aX+b). A positive *a* value indicates an increasing trend of annual WUE, and vice versa. The trends of WUE were examined for the entire country, each vegetation type, and each sub-region, respectively.

We also assessed the responses of WUE to drought. The widely used drought indicator in agricultural, hydrological, and ecological studies, standardized precipitation index (SPI), was used to measure the severity and duration of drought. One unique feature of SPI is that it can successively monitor spatial drought conditions at flexible time scales^57^. The 12-month SPIs through the end of December for a given year was used to define drought severity^28^. The 1, 3, 6, 9, and 12 month SPI was used to evaluate the effects of drought on WUE.

## Additional Information

**How to cite this article**: Liu, Y. *et al.* Water use efficiency of China's terrestrial ecosystems and responses to drought. *Sci. Rep.* **5**, 13799; doi: 10.1038/srep13799 (2015).

## Supplementary Material

supplementary tables and figures
