# Supplementary material for: Water use efficiency of China’s terrestrial ecosystems and responses to drought
Source: Sci Rep. 2015 Sep 8;5:13799. doi: 10.1038/srep13799 (PMC4562296; doi:10.1038/srep13799)
Supplement: supplementary tables and figures [file srep13799-s1.doc]

##### Water use efficiency of China’s terrestrial ecosystems and responses to drought

Yibo Liu^1, 2, 3^, Jingfeng Xiao^4, 5^, Weimin Ju2, 3[[1]](#footnote-2)*, Yanlian Zhou^2, 6^, Shaoqiang Wang^7^ & Xiaocui Wu^2, 3^

^1^ Jiangsu Key Laboratory of Agricultural Meteorology, School of Applied Meteorology, Nanjing University of Information Science and Technology, Nanjing, 210044, China, ^2^ Jiangsu Provincial Key Laboratory of Geographic Information Science and Technology, Nanjing University, Nanjing, 210023, China, ^3^ International Institute for Earth System Sciences, Nanjing University, Nanjing, 210023, China, ^4^Earth Systems Research Center, Institute for the Study of Earth, Oceans, and Space, University of New Hampshire, Durham, NH 03824, USA, ^5^ International Center for Ecology, Meteorology, and Environment, School of Applied Meteorology, Nanjing University of Information Science and Technology, Nanjing, 210044, China, ^6^ School of Geographic and Oceanographic Sciences, Nanjing University, Nanjing, 210023, China, ^7^ Key Laboratory of Ecosystem Network Observation and Modeling, Institute of Geographic Sciences and Natural Resources Research, Chinese Academy of Sciences, Beijing, 100101, China

##### List of Tables

Table S1. Values of key biochemical and biophysical parameters used for different vegetation types in this study.

Table S2 Descriptions of EC flux sites used for model validation.

Table S1 Key biochemical and biophysical parameter values used for different vegetation types used in this study. (evergreen needleleaf forests (ENF), deciduous needleleaf forests (DNF), evergreen broadleaf forests (EBF), deciduous broadleaf forests (DBF), mixed forests (MF), shrublands (SHR), croplands (CRO), grasslands (GRA), non-vegetation (NOV))

| Parameters | ENF | EBF | DNF | DBF | MF | SHR | GRA | CRO |
| --- | --- | --- | --- | --- | --- | --- | --- | --- |
| Clumping index ^a^ | 0.6[^1^](#_ENREF_1) | 0.8[^1^](#_ENREF_1) | 0.6[^1^](#_ENREF_1) | 0.8[^1^](#_ENREF_1) | 0.7[^2^](#_ENREF_2) | 0.5[^1^](#_ENREF_1) | 0.85[^3^](#_ENREF_3) | 0.85[^3^](#_ENREF_3) |
| Maximum stomatal conductance ^b^ | 2.0[^3^](#_ENREF_3) | 4.0[^3^](#_ENREF_3) | 2.5[^3^](#_ENREF_3) | 4.5[^3^](#_ENREF_3) | 3.6[^3^](#_ENREF_3) | 4.0[^2^](#_ENREF_2) | 10[^3^](#_ENREF_3) | 10[^3^](#_ENREF_3) |
| Maximum carboxylation at 25 °C ^C^ | 20[^3^](#_ENREF_3) | 32[^3^](#_ENREF_3) | 30[^3^](#_ENREF_3) | 25[^3^](#_ENREF_3) | 26[^3^](#_ENREF_3) | 33[^4^](#_ENREF_4) | 21[^3^](#_ENREF_3) | 96[^3^](#_ENREF_3) |
| Speciﬁc leaf area ^d^ | 20[^1^](#_ENREF_1) | 25[^3^](#_ENREF_3) | 22.5[^3^](#_ENREF_3) | 26.5[^3^](#_ENREF_3) | 24[^3^](#_ENREF_3) | 28.7[^3^](#_ENREF_3) | 30[^2^](#_ENREF_2) | 30[^2^](#_ENREF_2) |
| Leaf respiration coefficient ^e^ | 0.0022[^1^](#_ENREF_1) | 0.0042[^3^](#_ENREF_3) | 0.0024[^3^](#_ENREF_3) | 0.0048[^3^](#_ENREF_3) | 0.005[^3^](#_ENREF_3) | 0.006[^1^](#_ENREF_1) | 0.0007[^1^](#_ENREF_1) | 0.0008[^1^](#_ENREF_1) |
| Stem respiration coefficient ^e^ | 0.001[^1^](#_ENREF_1) | 0.001[^1^](#_ENREF_1) | 0.001[^5^](#_ENREF_5) | 0.001[^5^](#_ENREF_5) | 0.001[^1^](#_ENREF_1) | 0.001[^1^](#_ENREF_1) | 0.001[^5^](#_ENREF_5) | 0.001[^5^](#_ENREF_5) |
| Coarse root respiration coefficient ^e^ | 0.001[^1^](#_ENREF_1) | 0.001[^1^](#_ENREF_1) | 0.001[^1^](#_ENREF_1) | 0.001[^1^](#_ENREF_1) | 0.001[^1^](#_ENREF_1) | 0.001[^1^](#_ENREF_1) | 0.0015[^1^](#_ENREF_1) | 0.0015[^1^](#_ENREF_1) |
| Fine root respiration coefficient ^e^ | 0.00275[^3^](#_ENREF_3) | 0.0036[^3^](#_ENREF_3) | 0.0036[^3^](#_ENREF_3) | 0.0036[^3^](#_ENREF_3) | 0.0036[^3^](#_ENREF_3) | 0.003[^1^](#_ENREF_1) | 0.003[^1^](#_ENREF_1) | 0.003[^1^](#_ENREF_1) |
| Q10 for leaf ^a^ | 2.1[^1^](#_ENREF_1) | 2.1[^1^](#_ENREF_1) | 2.1[^1^](#_ENREF_1) | 2.1[^1^](#_ENREF_1) | 2.1[^1^](#_ENREF_1) | 2.1[^1^](#_ENREF_1) | 2.1[^1^](#_ENREF_1) | 2.1[^1^](#_ENREF_1) |
| Q10 for stem ^a^ | 1.5[^1^](#_ENREF_1) | 2.0[^1^](#_ENREF_1) | 2.0[^1^](#_ENREF_1) | 2.0[^1^](#_ENREF_1) | 2.0[^1^](#_ENREF_1) | 2.0[^1^](#_ENREF_1) | 1.5[^1^](#_ENREF_1) | 1.5[^1^](#_ENREF_1) |
| Q10 for root ^a^ | 1.9[^1^](#_ENREF_1) | 1.9[^1^](#_ENREF_1) | 1.9[^1^](#_ENREF_1) | 1.9[^1^](#_ENREF_1) | 1.9[^1^](#_ENREF_1) | 1.9[^1^](#_ENREF_1) | 1.9[^1^](#_ENREF_1) | 1.9[^1^](#_ENREF_1) |
| Leaf water potential at stomatal closure ^f^ | 2.3[^1^](#_ENREF_1) | 3.9[^1^](#_ENREF_1) | 2.3[^1^](#_ENREF_1) | 2.1[^1^](#_ENREF_1) | 2.3[^1^](#_ENREF_1) | 4.2[^1^](#_ENREF_1) | 2.7[^1^](#_ENREF_1) | 2.7[^1^](#_ENREF_1) |
| Snowmelt temperature coefficient ^g^ | 0.0022[^1^](#_ENREF_1) | 0.001[^1^](#_ENREF_1) | 0.0022[^1^](#_ENREF_1) | 0.001[^1^](#_ENREF_1) | 0.0015[^1^](#_ENREF_1) | 0.002[^1^](#_ENREF_1) | 0.001[^1^](#_ENREF_1) | 0.001[^1^](#_ENREF_1) |

a: No unit; b: mm s^-1^; c: umol CO_2_ m^-2^ s^-1^; d: m^2^ kg^-1^ C; e: kg C d^-1^ kg^-1^; f: -M pa; g: m d^-1^ °C^-1^.

Table S2. Descriptions of eddy covariance (EC) flux sites used for model validation.

| Ecosystem type | Site | Latitude (°N) | Longitude (°E) | Period | Measured WUE (GPP/ET) (g C kg^-1^ H_2_O) | Reference |
| --- | --- | --- | --- | --- | --- | --- |
| Forest | Changbaishan | 42.40 | 128.08 | 2003-2005 | 2.57±0.35 | [^6^](#_ENREF_6) |
|  | Qianyanzhou | 26.73 | 115.05 | 2003-2005 | 2.53±0.48 | [^6^](#_ENREF_6) |
|  | Daxing | 39.53 | 116.25 | 2007-2008 | 2.42±0.19 | [^7^](#_ENREF_7) |
|  | Dinghushan | 23.17 | 112.53 | 2003-2005 | 2.30±0.15 | [^8^](#_ENREF_8) |
|  | Yueyang | 29.31 | 112.51 | 2006 | 2.26 | [^9^](#_ENREF_9) |
|  | Ailaoshan | 24.53 | 101.02 | 2010 | 2.32 | [^10^](#_ENREF_10) |
|  | Xishuangbanna | 21.95 | 101.20 | 2003-2006 | 2.27±0.07 | [^11^](#_ENREF_11) [^12^](#_ENREF_12) |
|  | Huitong | 26.83 | 109.75 | 2009 | 2.36 | [^13^](#_ENREF_13)  [^14^](#_ENREF_14) |
|  | Lin’an | 30.18 | 119.34 | 2011 | 1.84 |  |
|  | Hunang | 33.00 | 117.00 | 2005 | 1.85 | [^10^](#_ENREF_10) |
|  | Xiaolangdi | 35.02 | 112.47 | 2007-2009 | 2.41±0.07 | [^17^](#_ENREF_17) |
|  | Kubuqi forest | 40.54 | 108.69 | 2006 | 0.66 | [^10^](#_ENREF_10) |
|  | Laoshan | 45.33 | 127.67 | 2004-2006 | 3.89±0.58 |  |
| Grassland | Shidi | 37.61 | 101.33 | 2003-2005 | 0.71±0.04 | [^20^](#_ENREF_20) |
|  | Gancaitan | 37.66 | 101.33 | 2003-2005 | 1.26±0.13 | [^20^](#_ENREF_20) |
|  | Dangxiong 1 | 30.85 | 91.08 | 2003-2005 | 0.41±0.01 | [^20^](#_ENREF_20) |
|  | Neimeng | 43.55 | 116.66 | 2003-2005 | 0.88±0.44 | [^20^](#_ENREF_20) |
|  | Changling | 44.58 | 123.50 | 2007-2008 | 1.59±0.44 | [^21^](#_ENREF_21) |
|  | Duolun grassland | 42.05 | 116.28 | 2006-2007 | 0.86±0.15 | [^22^](#_ENREF_22) |
|  | Xilinhot1 | 44.13 | 116.33 | 2004-2006 | 0.47±0.11 | [^23^](#_ENREF_23) |
|  | Dangxiong2 | 29.67 | 91.33 | 2004-2008 | 0.36±0.07 | [^10^](#_ENREF_10) |
|  | Sanjiangyuan | 34.35 | 100.55 | 2006 | 1.02 | [^24^](#_ENREF_24) [^25^](#_ENREF_25) |
|  | Haibei | 37.62 | 101.30 | 2002-2004 | 1.63±0.06 | [^26^](#_ENREF_26) [^27^](#_ENREF_27) |
|  | Xilinhot2 | 43.55 | 116.67 | 2006 | 0.66 | [^10^](#_ENREF_10) |
|  | Xilinhot3 | 43.55 | 116.67 | 2006 | 0.53 | [^10^](#_ENREF_10) |
|  | Fukang | 44.28 | 87.93 | 2004-2007 | 1.10±0.51 |  |
|  | Tongyu grassland | 44.59 | 122.52 | 2003-2008 | 0.94±0.28 | [^30^](#_ENREF_30) [^31^](#_ENREF_31) |
| Cropland | Duolun cropland | 42.05 | 116.28 | 2006-2007 | 1.06±0.01 | [^22^](#_ENREF_22) |
|  | Yucheng | 36.83 | 116.57 | 2003-2008 | 2.96±0.10 | [^10^](#_ENREF_10) |
|  | Weishan | 36.65 | 116.05 | 2005-2007 | 3.12±0.15 | [^32^](#_ENREF_32) [^33^](#_ENREF_33) |
|  | Yingke | 38.86 | 100.41 | 2008 | 2.18 | [^10^](#_ENREF_10) |
|  | Tongyu crop | 44.57 | 122.92 | 2003-2008 | 0.93±0.18 | [^30^](#_ENREF_30) [^31^](#_ENREF_31) |
| Wetland | Gaoqiao | 21.57 | 109.76 | 2010 | 2.01 | [^34^](#_ENREF_34) |
|  | Yunxia | 23.92 | 117.42 | 2010 | 2.11 | [^34^](#_ENREF_34) |
|  | Panjin | 41.15 | 121.92 | 2005 | 3.01 | [^35^](#_ENREF_35) [^36^](#_ENREF_36) |

##### List of Figures

Figure S1．The variations of annual WUE for China and its nine sub-regions over the period from 2000 to 2011. (R1: Northeast China, R2: Inner Mongolia, R3: Northwest China, R4: North China, R5: Central China, R6: Tibet Plateau, R7: Southeast China, R8: South China, and R9: Southwest China).

Figure S2．Trends of annual mean WUE averaged for different vegetation types: ENF-evergreen needleleaf forests; EBF-evergreen broadleaf forests; DNF-deciduous needleleaf forests; DBF-deciduous broadleaf forests; MF-mixed forests; SHR-shrublands; GRA-grasslands; CRO-croplands.

Figure S3．Relationships between annual mean LAI and annual WUE for different vegetation types in China. The full names of the vegetation types are provided in Fig. S2.

Figure S4．Relationships between annual precipitation and annual WUE for different vegetation types in China. The full names of the vegetation types are provided in Fig. S2.

Figure S5．The relative departures of WUE, NPP, and ET and correlations of WUE with NPP and ET for China and its nine sub-regions. The full names of sub-regions are provided in Fig. S1.

Figure S6. Spatial distribution of mean annual WUE and trends based on BEPS simulation and MODIS NPP/ET products during the period from 2000 to 2011: (a) mean annual WUE simulated by BEPS; (b) mean annual WUE derived from MODIS NPP and ET products; (c) trends of annual WUE simulated by BEPS; (d) trends of annual WUE derived from MODIS NPP and ET products. This figure was produced using ArcGIS 10.0.

Figure S7．Changes of NPP/T (a) and T/ET (b) over China during the period from 2000 to 2011. This figure was produced using ArcGIS 10.0.

Figure S8. Relative changes (%) of simulated national total NPP, ET and WUE relative to the corresponding values simulated using undisturbed inputs for different sensitivity scenarios of LAI, precipitation, and temperature.

Figure S9. Comparisons of daily radiation in 2005 interpolated using IDW method with EC tower measurements at 8 ChinaFLUX sites.

Figure S10. Comparisons of daily temperature in 2005 interpolated using IDW method with EC tower measurements at 8 ChinaFLUX sites.

Figure S11. Comparisons of daily precipitation in 2005 interpolated using IDW method with EC tower measurements at 8 ChinaFLUX sites.

Figure S12 Annual mean temperature and annual precipitation of China in 2000: (a) temperature interpolated using the IDW method; (b) temperature provided by CMA; (c) precipitation interpolated using the IDW method ; (d) precipitation provided by CMA. This figure was produced using ArcGIS 10.0.

Figure S13．Validation of BEPS simulated annual WUE* (=GPP/ET) against WUE* derived from EC measurements. The error bars stand for the standard deviation of WUE*.

| 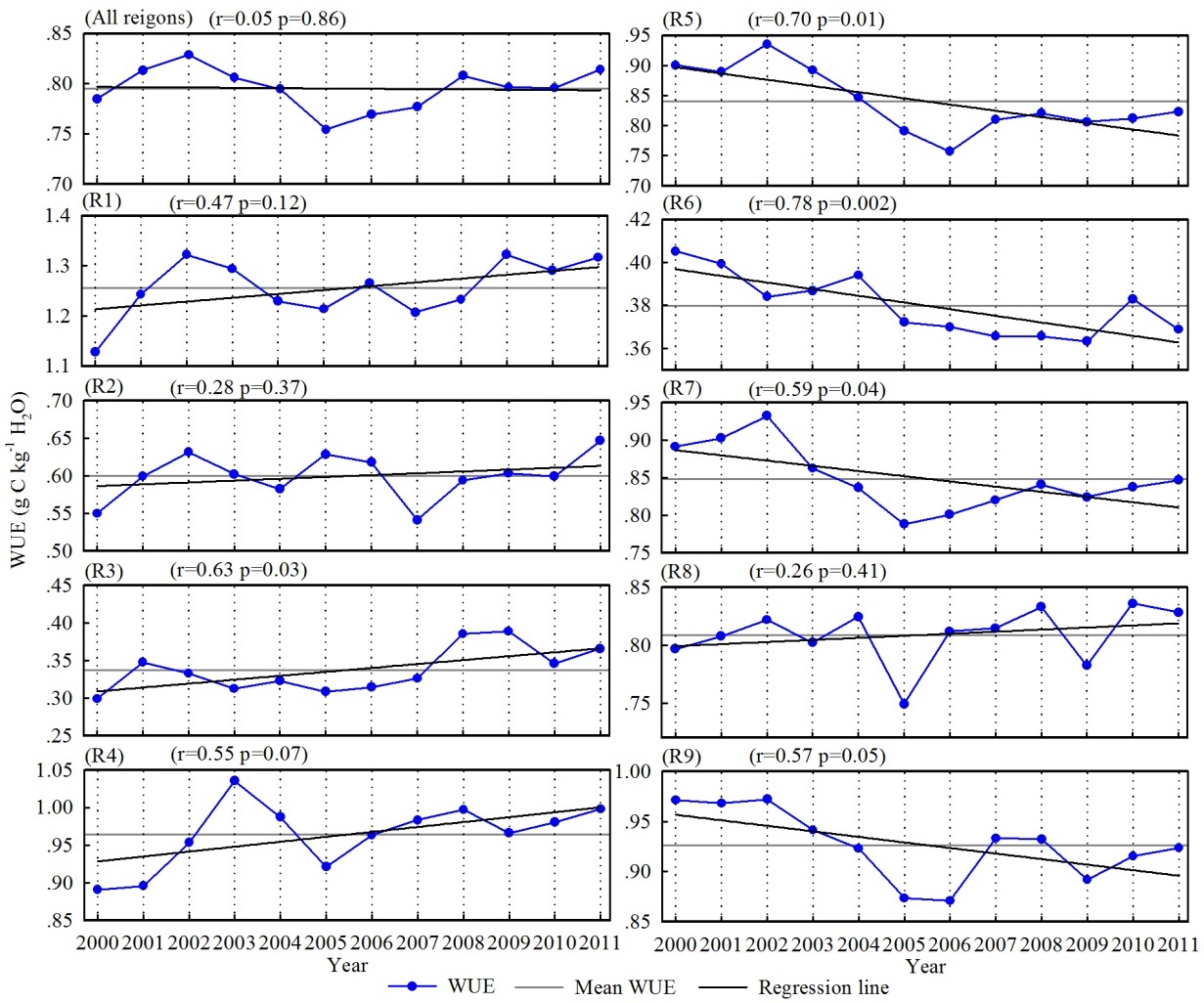 |
| --- |
| Figure S1．The variations of annual WUE for China and its nine sub-regions over the period from 2000 to 2011. (R1: Northeast China, R2: Inner Mongolia, R3: Northwest China, R4: North China, R5: Central China, R6: Tibet Plateau, R7: Southeast China, R8: South China, and R9: Southwest China). |

| 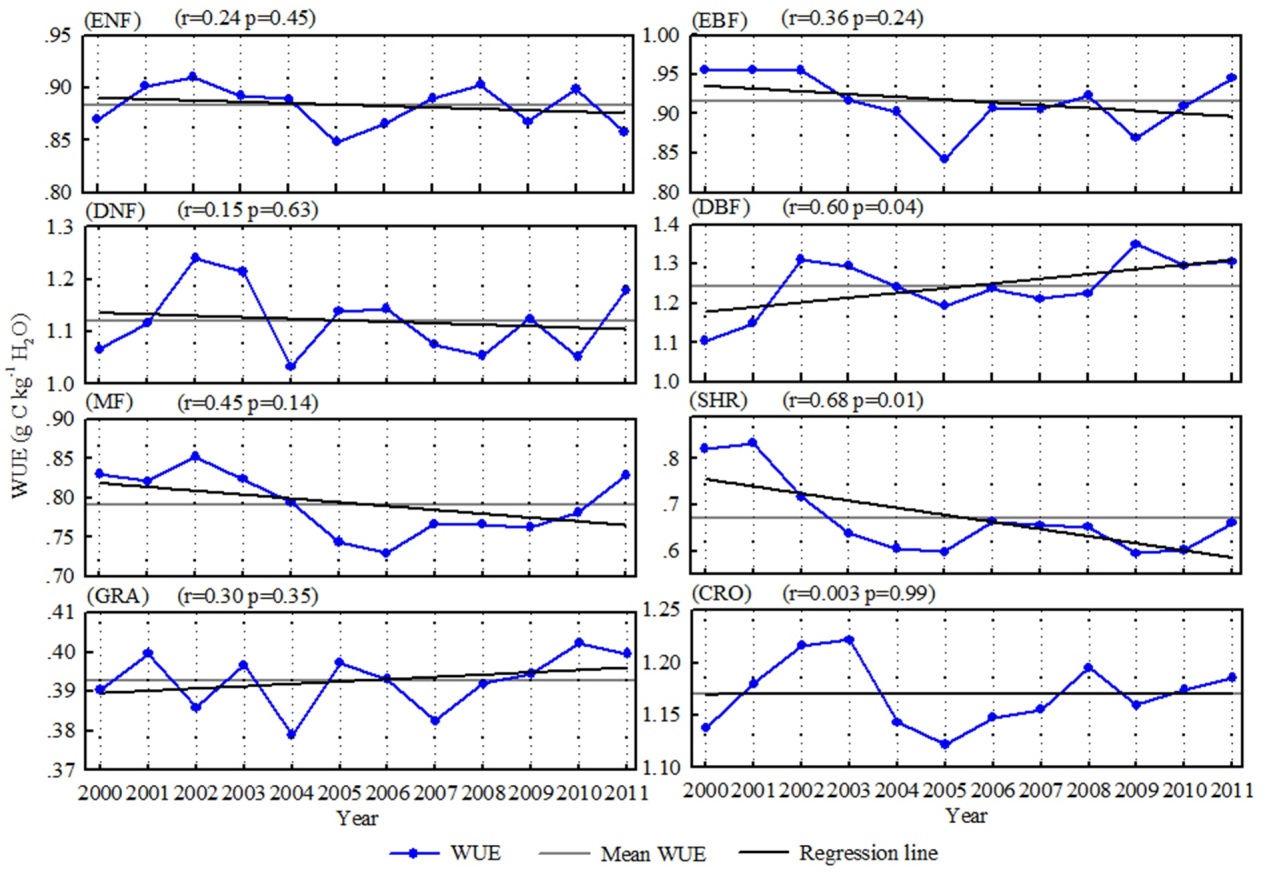 |
| --- |
| Figure S2．Trends of annual mean WUE averaged for different vegetation types: ENF-evergreen needleleaf forests; EBF-evergreen broadleaf forests; DNF-deciduous needleleaf forests; DBF-deciduous broadleaf forests; MF-mixed forests; SHR-shrublands; GRA-grasslands; CRO-croplands. |

| 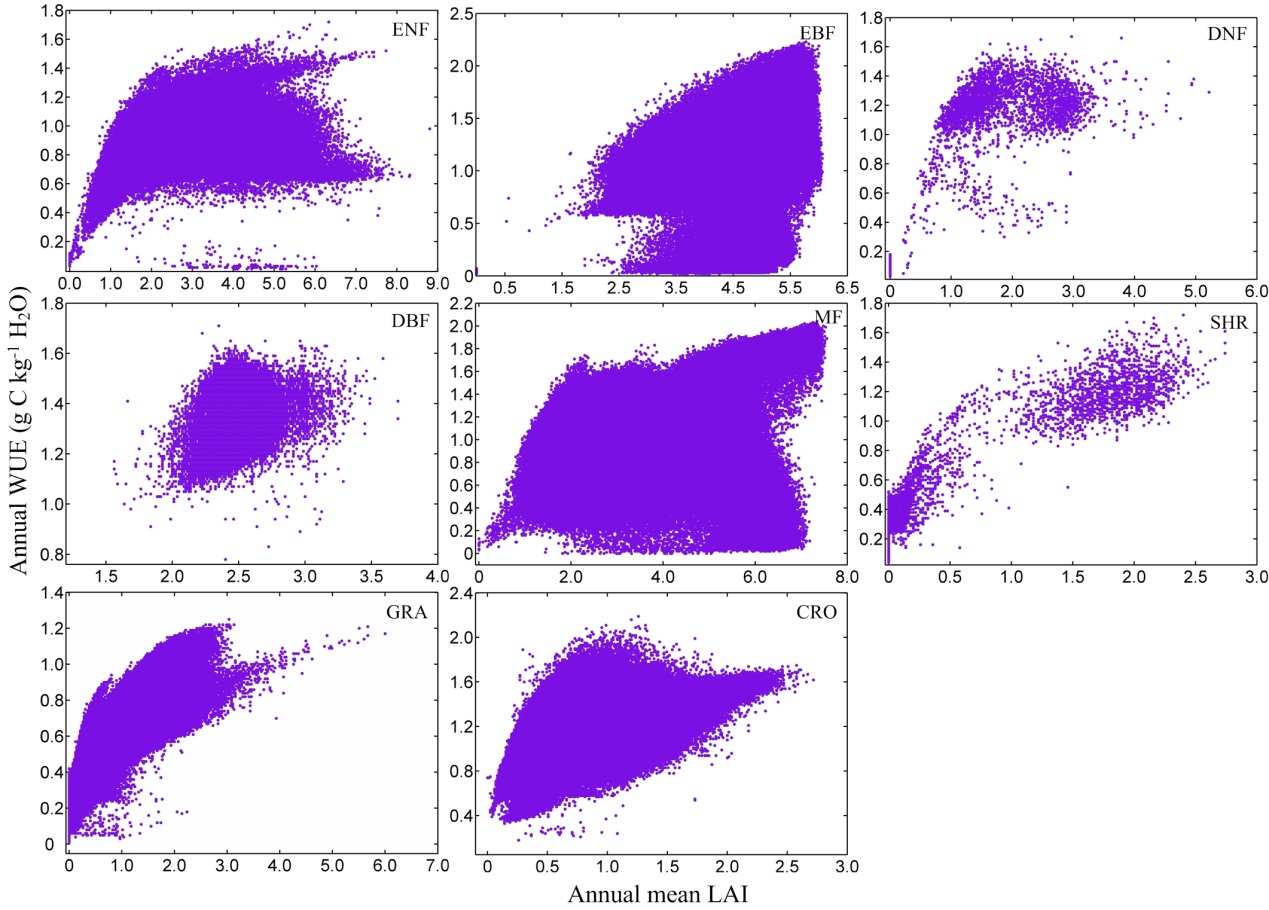 |
| --- |
| Figure S3．Relationships between annual mean LAI and annual WUE for different vegetation types in China. The full names of the vegetation types are provided in Fig. S2. |

| 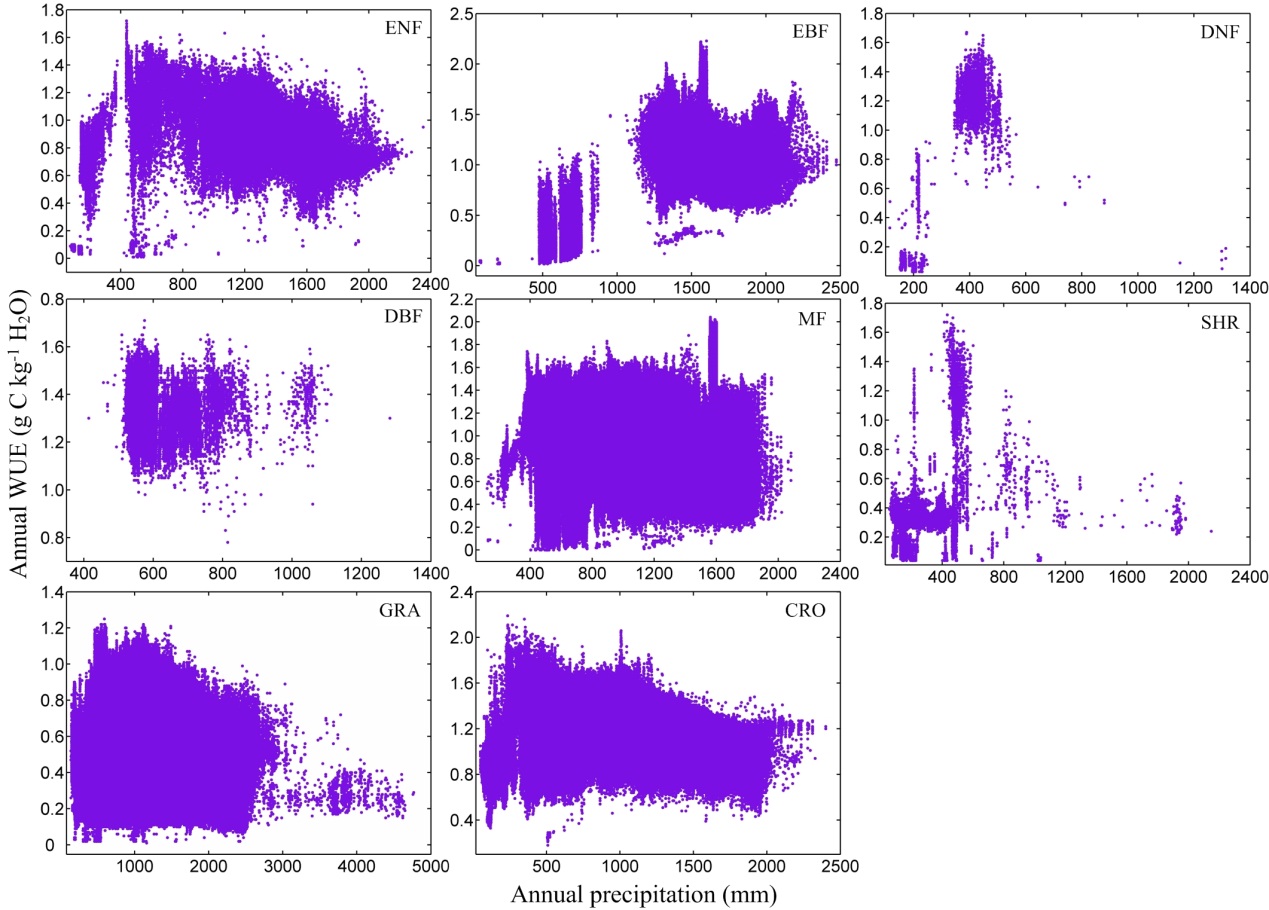 |
| --- |
| Figure S4．Relationships between annual precipitation and annual WUE for different vegetation types in China. The full names of the vegetation types are provided in Fig. S2. |

| 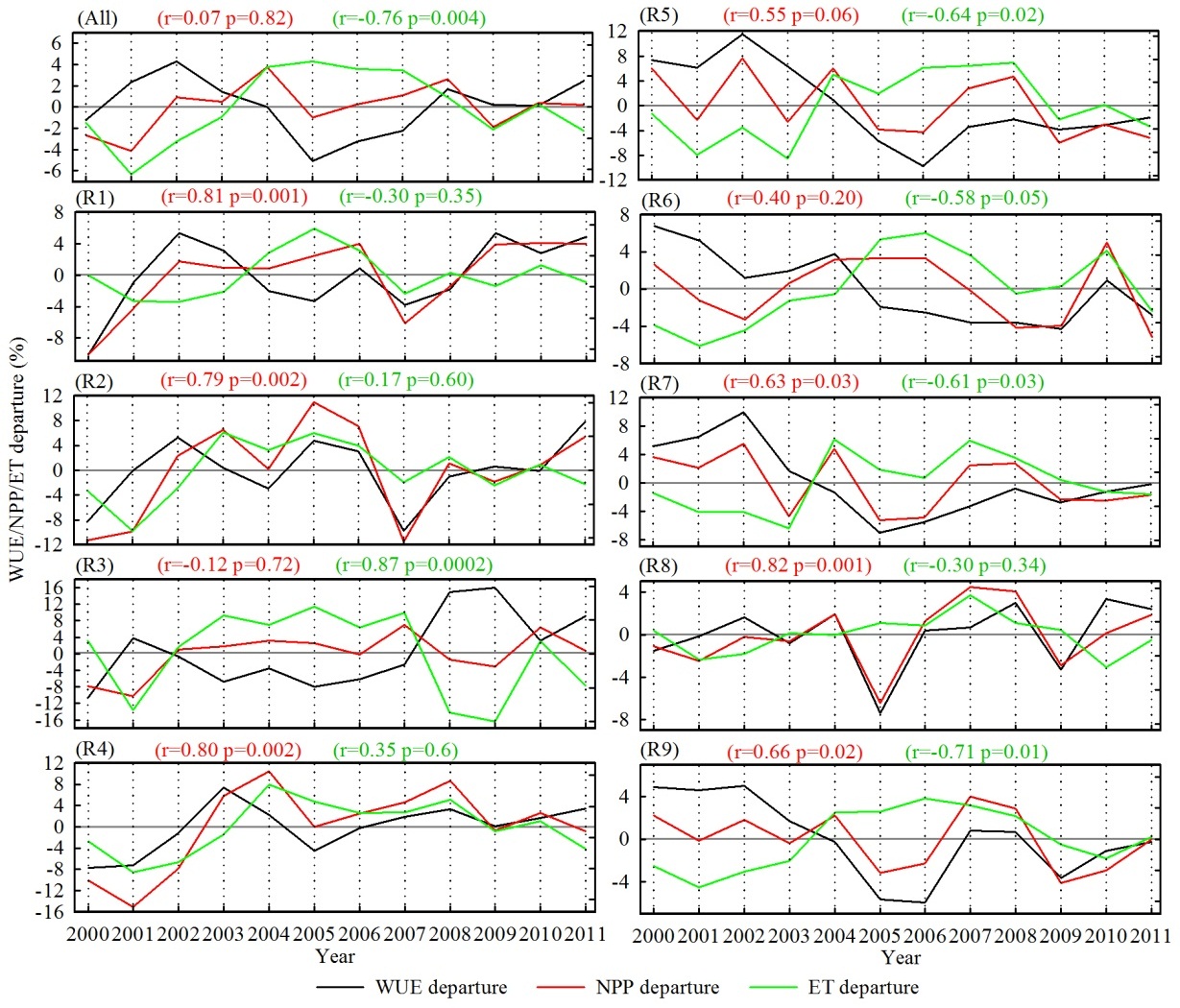 |
| --- |
| Figure S5．The relative departures of WUE, NPP, and ET and correlations of WUE with NPP and ET for China and tis nine sub-regions. The full names of sub-regions are provided in Fig. S1. |

| 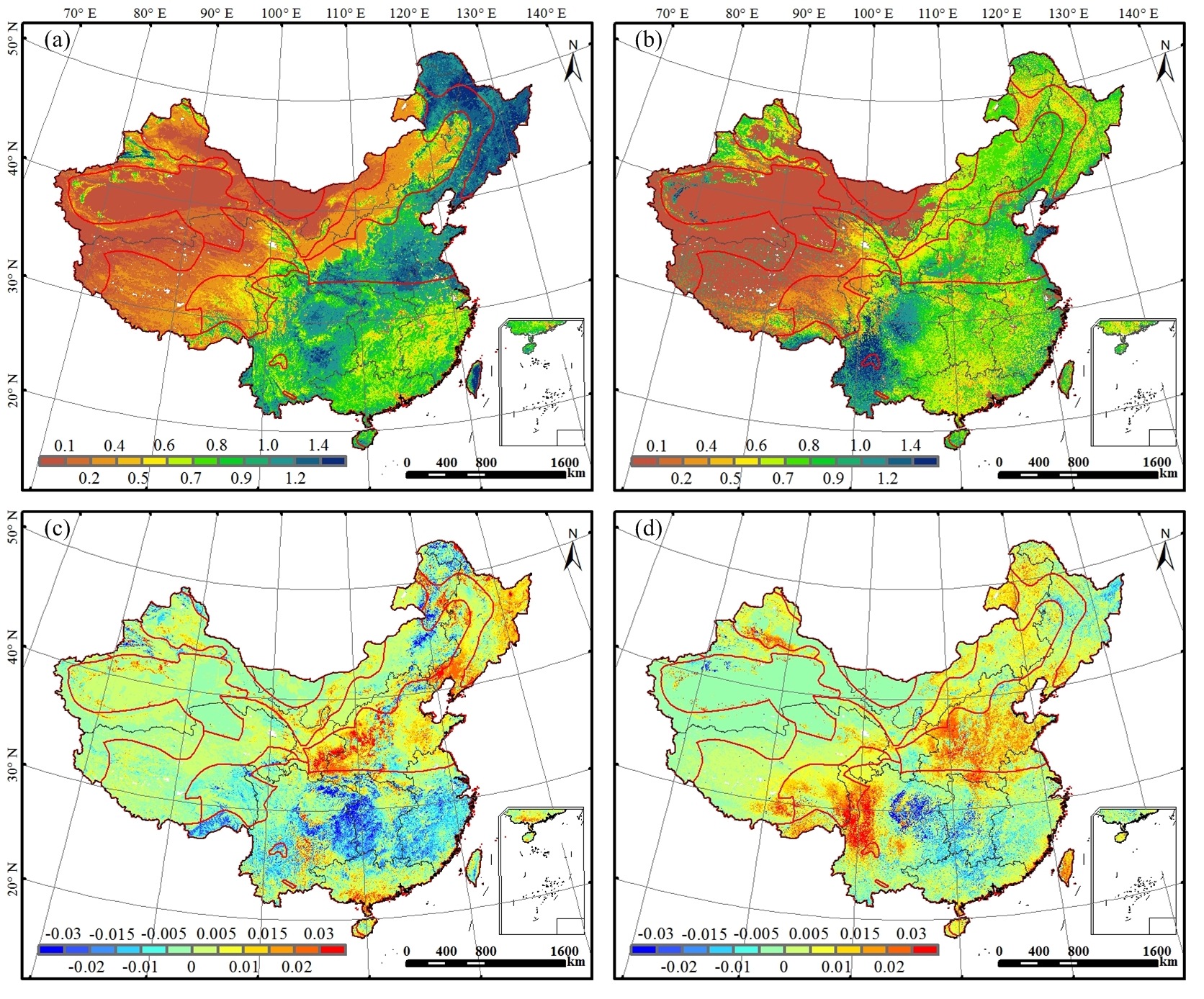 |
| --- |
| Figure S6. Spatial distribution of mean annual WUE and trends based on BEPS simulation and MODIS NPP/ET products during the period from 2000 to2011: (a) mean annual WUE simulated by BEPS; (b) mean annual WUE derived from MODIS NPP and ET products; (c) trends of annual WUE simulated by BEPS; (d) trends of annual WUE derived from MODIS NPP and ET products. This figure was produced using ArcGIS 10.0. |

| 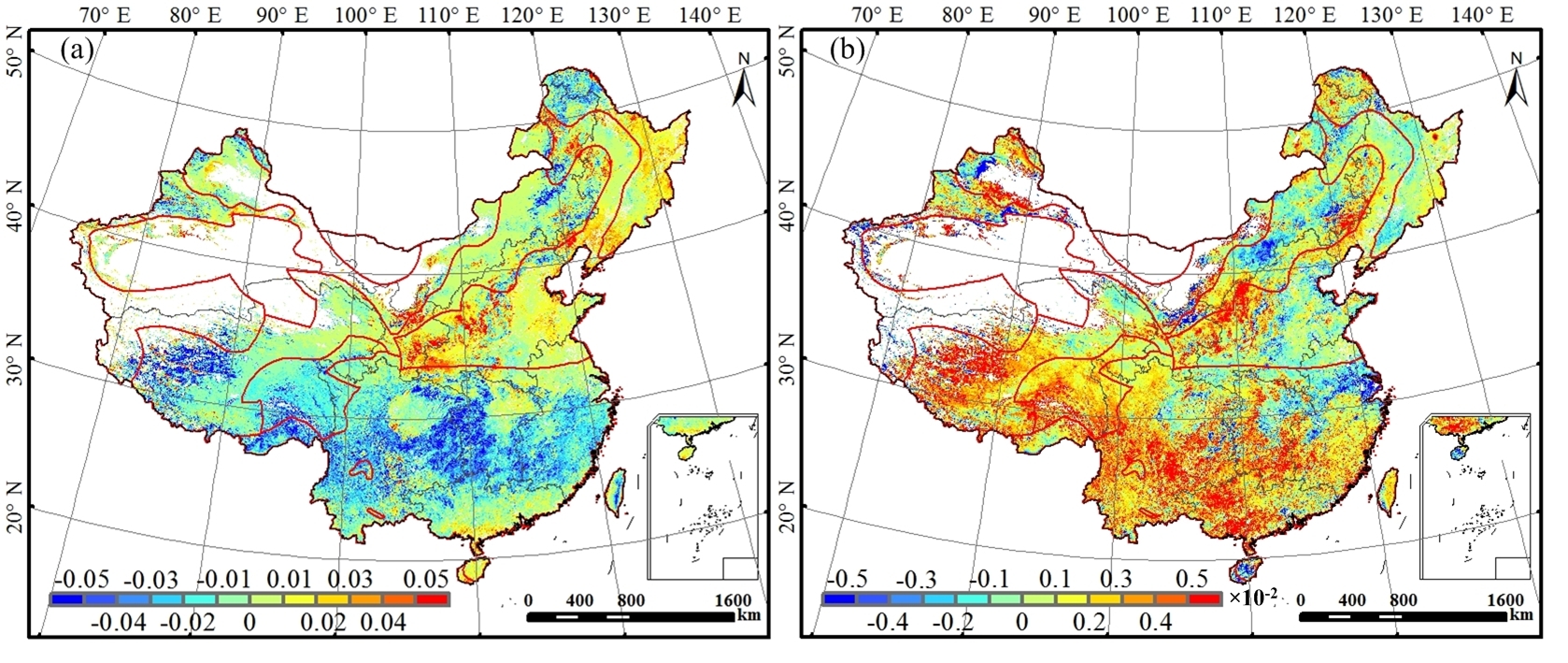 |
| --- |
| Figure S7．Changes of NPP/T (a) and T/ET (b) over China during the period from 2000 to 2011. This figure was produced using ArcGIS 10.0. |

| 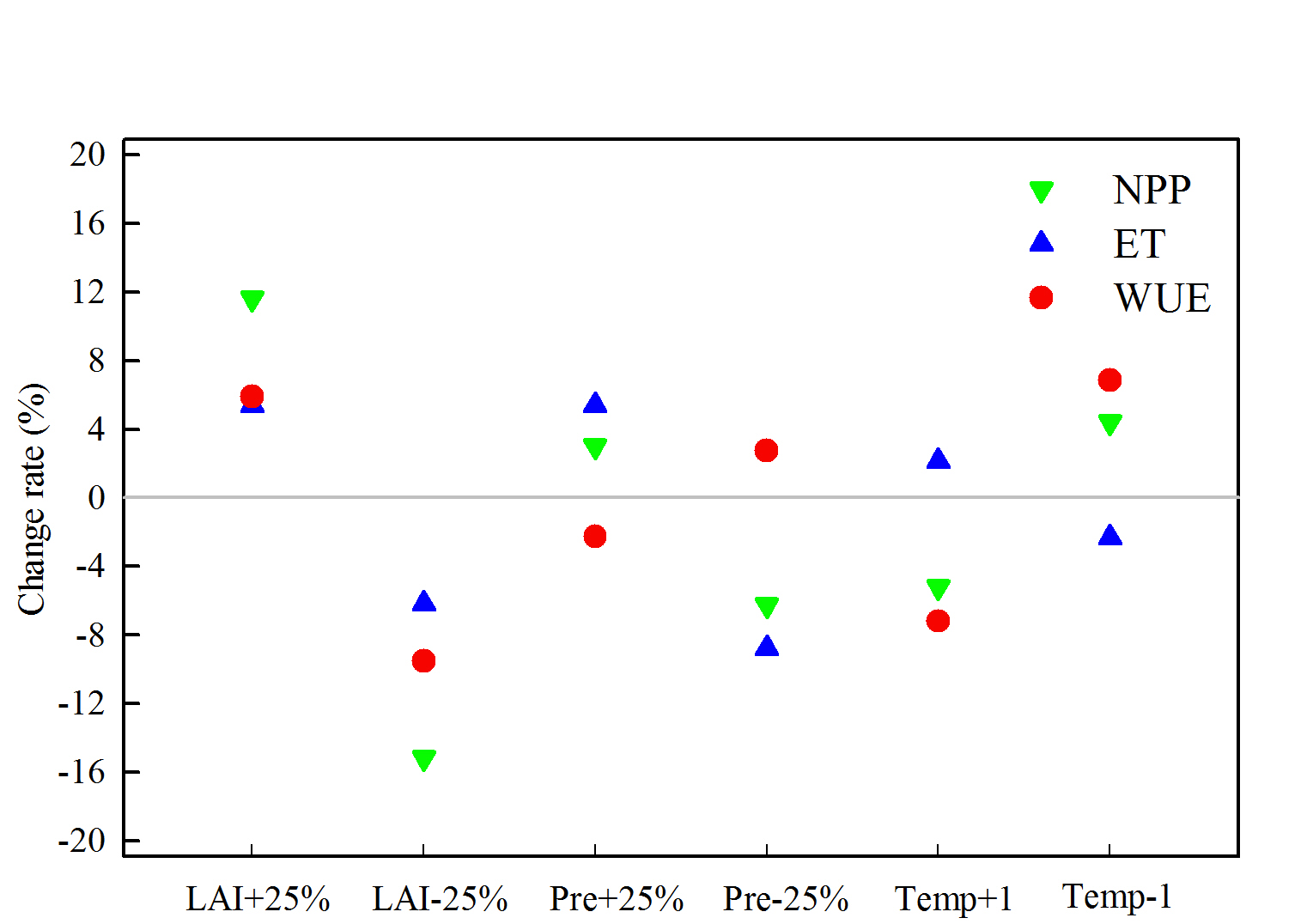 |
| --- |
| Figure S8. Relative changes (%) of simulated national total NPP, ET and WUE relative to the corresponding values simulated using undisturbed inputs for different sensitivity scenarios of LAI, precipitation, and temperature. |

| 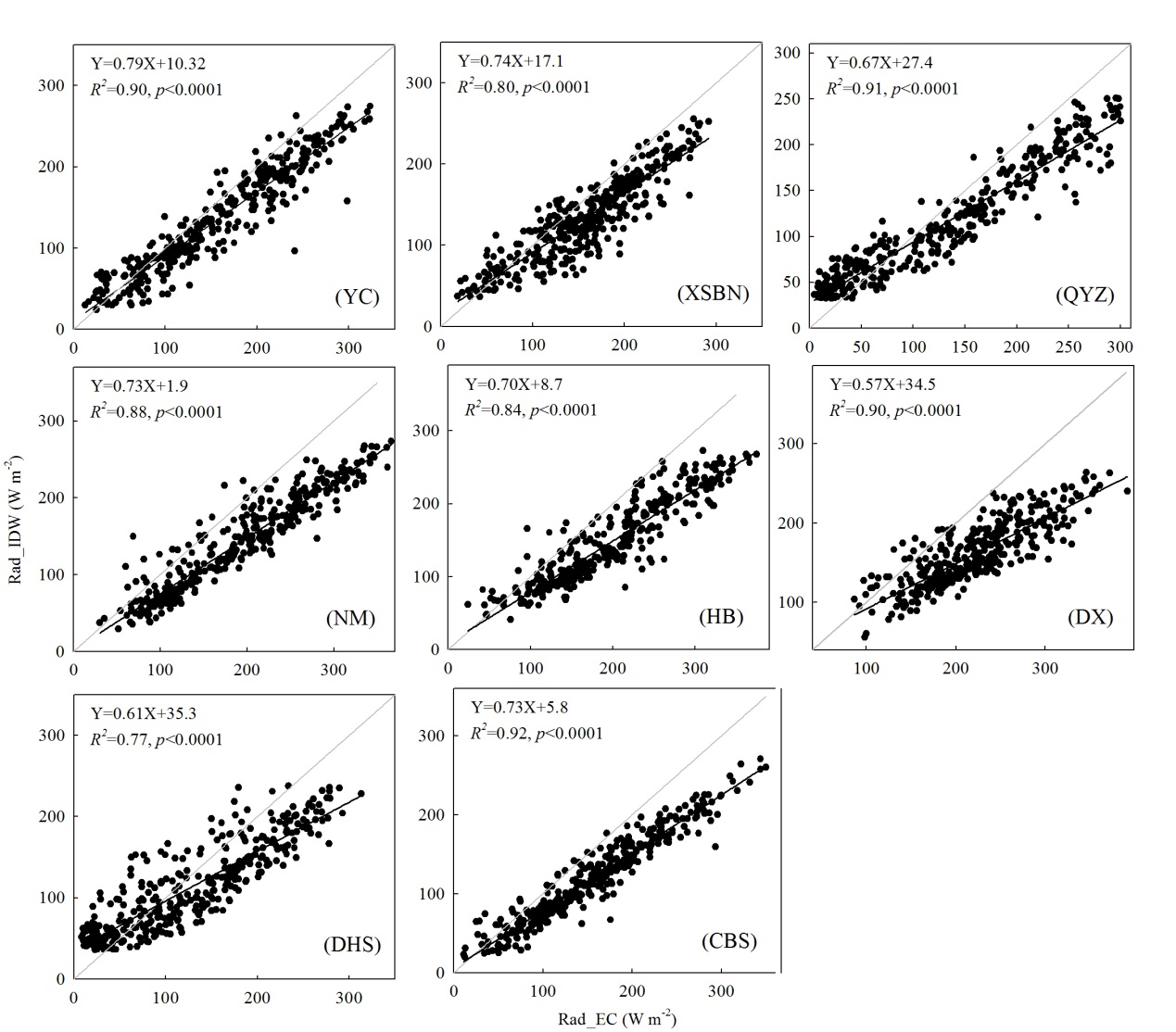 |
| --- |
| Figure S9 Comparisons of daily radiation in 2005 interpolated using IDW method with EC tower measurements at 8 ChinaFLUX sites. |

| 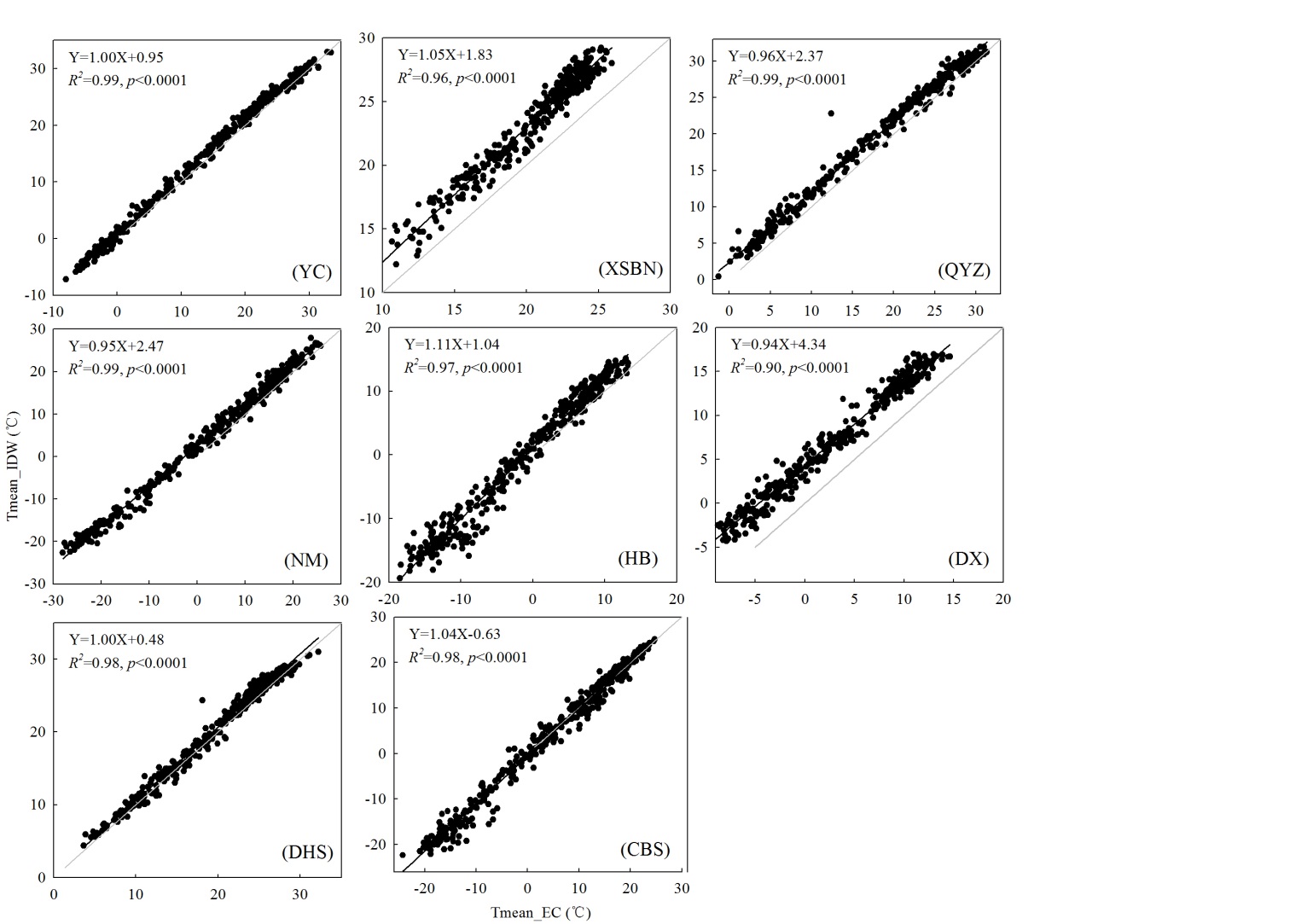 |
| --- |
| Figure S10Comparisons of daily temperature in 2005 interpolated using IDW method with EC tower measurements at 8 ChinaFLUX sites. |

| 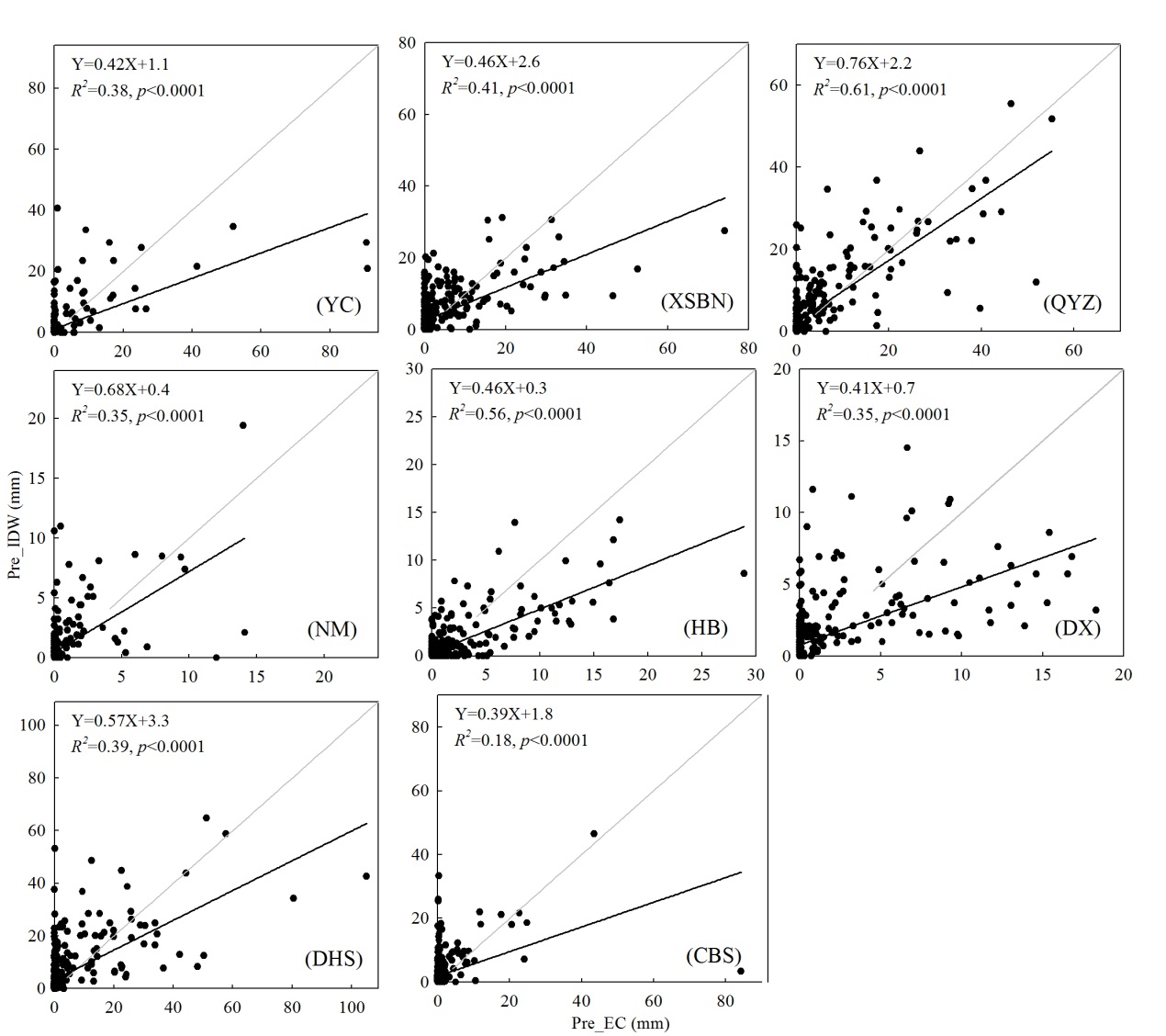 |
| --- |
| Figure S11. Comparisons of daily precipitation in 2005 interpolated using IDW method with EC tower measurements at 8 ChinaFLUX sites. |

| 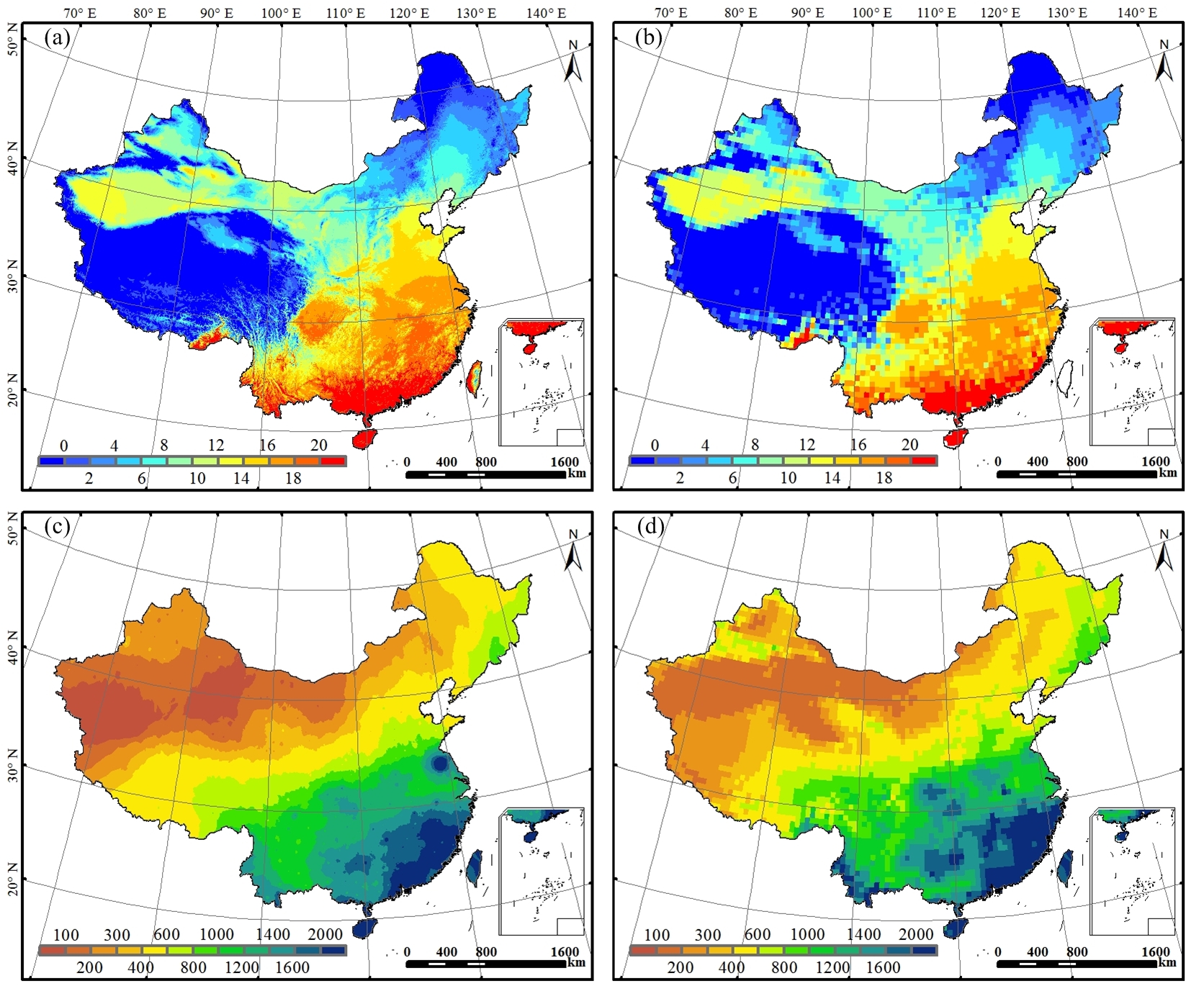 |
| --- |
| Figure S12 Annual mean temperature and annual precipitation of China in 2000: (a) temperature interpolated using the IDW method; (b) temperature provided by CMA; (c) precipitation interpolated using the IDW method ; (d) precipitation provided by CMA. This figure was produced using ArcGIS 10.0. |

| 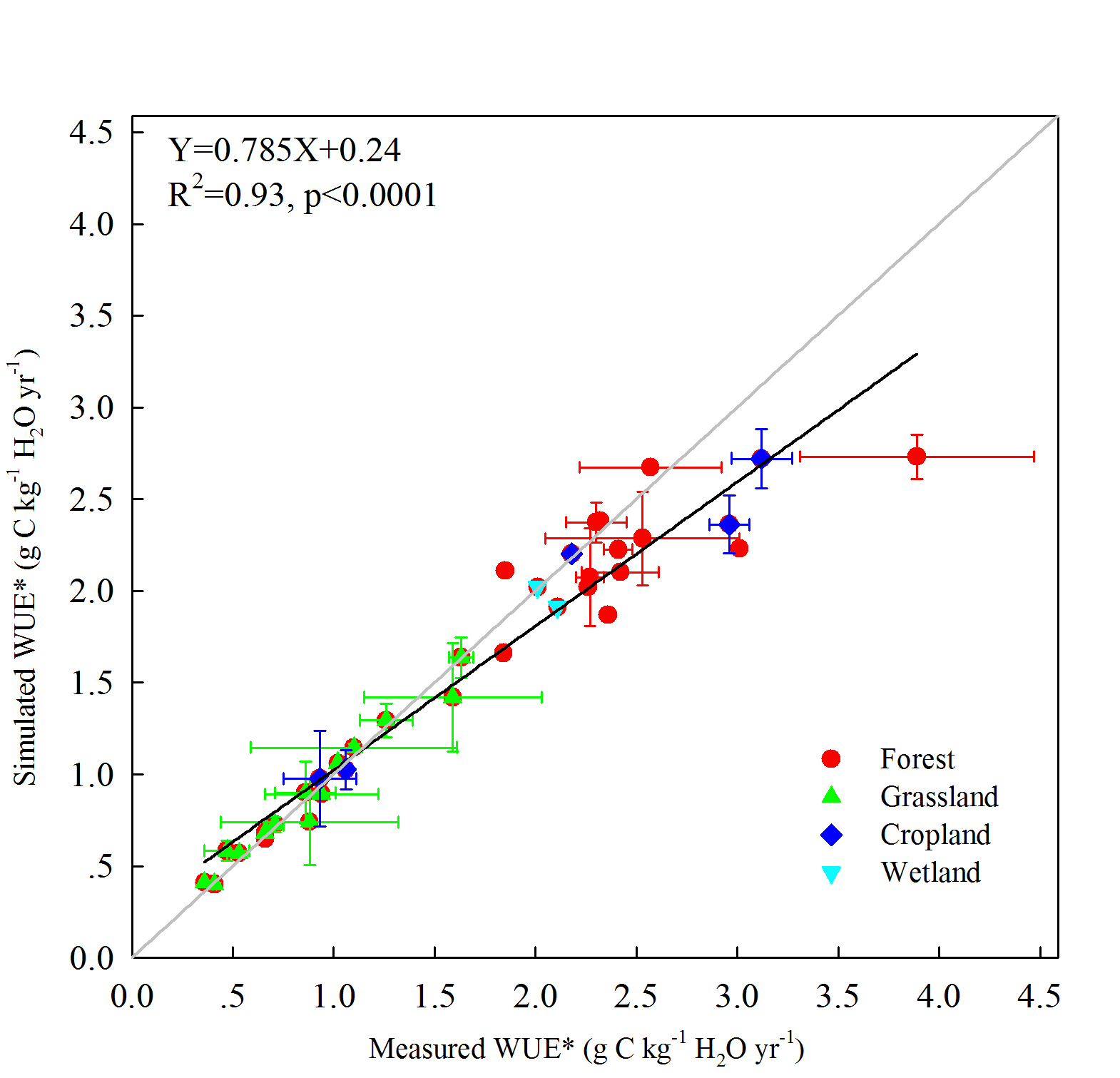 |
| --- |
| Figure S13．Validation of BEPS simulated annual WUE*(=GPP/ET) against WUE* derived from EC measurements. The error bars stand for the standard deviation of WUE*. |

**Reference**

1. Zhang, F. *et al.* Variations of terrestrial net primary productivity in East Asia. *Terr. Atmos. Ocean. Sci*. **23**, 425-437 (2012).

2. Feng, X. *et al.* Net primary productivity of China's terrestrial ecosystems from a process model driven by remote sensing. *J. Environ. Manage.* **85**, 563-573 (2007).

3. Liu, Y. *et al.* Changes of net primary productivity in China during recent 11 years detected using an ecological model driven by MODIS data. *Front. Earth Sci.* **7**, 112-127 (2013).

4. Gonsamo, A. *et al.* Improved assessment of gross and net primary productivity of Canada's landmass. *J. Geophys. Res.-Biogeosci.* **118**, 1546-1560 (2013).

5. Liu, J., Chen, J. M., Cihlar, J. & Park, W. M. A process-based boreal ecosystem productivity simulator using remote sensing inputs. *Remote Sens. Environ.* **62**, 158-175 (1997).

6. Yu, G. R. *et al.* Water-use efficiency of forest ecosystems in eastern China and its relations to climatic variables. *New Phytol.* **177**, 927-937 (2008).

7. Liu, C. *et al.* Quantifying evapotranspiration and biophysical regulations of a poplar plantation assessed by eddy covariance and sap-flow methods. *J. Plant. Ecol.* **33**, 706-718 (2009).

8. Zhou, G. *et al.* Quantifying the hydrological responses to climate change in an intact forested small watershed in Southern China. *Global. Change. Biol.* **17**, 3736-3746 (2011).

9. Wei, Y. *et al.* Source area in-FLUX measurements by FSAM model over the *Populus deltoides* plantation in Yueyang. *Scientia Silvae Sinicae*. **48**, 16-21 (2012).

10. Zhu, X. *et al.* Spatial variability of water use efficiency in China's terrestrial ecosystems. *Global Planet. Change*. **129**, 37-44 (2015).

11. Li, Z. H. *et al.* Evapotranspiration of a tropical rain forest in Xishuangbanna, southwest China. *Hydrol. Process.* **24**, 2405-2416 (2010).

12. Zhang, Y. P., Tan, Z. H., Song, Q. H., Yu, G. R. & Sun, X. M. Respiration controls the unexpected seasonal pattern of carbon flux in an Asian tropical rain forest. *Atmos. Environ.* **44**, 3886-3893 (2010).

13. Wang, W. *et al.* Characteristics of latent heat flux over Cunninghamia lanceolata plantations in Huitong county. *J. Cent. South Univer. For. & Tech.* **31**, 192-197 (2011).

14. Zhao, Z. *A study on carbon flux between Chinese Fir planations and atmosphere in subtropical belts* Doctor thesis, Central South University of Forestry and Technology, (2011).

15. Chen, Y., Jiang, H., Zhou, G., Shuang, Y. & Chen, J. Estimation of CO_2_ fluxes and its seasonal variations from the effective management Lei bamboo (*Phyllostachys Violascens*). *Acta Ecol. Sin.* **33**, 3434-3444 (2013).

16. Lin, E., Jiang, H. & Chen, Y. Water vapor flux variation and net radiation for a *Phyllostachys violascens* stand in Taihuyuan. *J. Zhejiang A.＆F. Univer.* **30**, 313-318 (2013).

17. Guo, L. *The variations of water use efficiency and evapotranspiration over a plantation in the southern part of hilly areas of North-China* Master thesis, Chinese Academy of Forestry, (2010).

18. Wang, H. *et al.* Carbon fluxes and their response to environmental variables in a Dahurian larch forest ecosystem in northeast China. *J. For. Res.* **19**, 1-10 (2008).

19. Cui, S. *Study on the CO_2_ Flux of a Larch Plantation in NE China by the micrometeorological method* Master thesis, Northeast Forestry University, (2007).

20. Hu, Z. M. *et al.* Effects of vegetation control on ecosystem water use efficiency within and among four grassland ecosystems in China. *Global. Change. Biol.* **14**, 1609-1619 (2008).

21. Dong, G. *et al.* Effects of spring drought on carbon sequestration, evapotranspiration and water use efficiency in the Songnen meadow steppe in Northeast China. *Ecohydrology*. **4**, 211-224 (2011).

22. Chen, S. P. *et al.* Energy balance and partition in Inner Mongolia steppe ecosystems with different land use types. *Agr. Forest. Meteorol.* **149**, 1800-1809 (2009).

23. Wang, Y. L., Zhou, G. S. & Wang, Y. H. Environmental effects on net ecosystem CO_2_ exchange at half-hour and month scales over Stipa krylovii steppe in northern China. *Agr. Forest. Meteorol.* **148**, 714-722 (2008).

24. Wu, L. *et al.* Variation in net CO_2_ exchange, gross primary production and its affecting factors in the planted pasture ecosystem in Sanjiangyuan Region of the Qinghai-Tibetan Plateau of China. *J. Plant. Ecol.* **34**, 770-780 (2010).

25. Li, J., Cai, H. & Cheng, Q. Characterizing the evapotranspiration of a degraded grassland in the Sanjiangyuan Region of Qinghai province. *Acta Prata. Sin.* **21**, 223-233 (2012).

26. Gu, S. *et al.* Characterizing evapotranspiration over a meadow ecosystem on the Qinghai-Tibetan Plateau. *J. Geophys. Res.* **113** (2008).

27. Kato, T. *et al.* Temperature and biomass influences on interannual changes in CO_2_ exchange in an alpine meadow on the Qinghai-Tibetan Plateau. *Global. Change. Biol.* **12**, 1285-1298 (2006).

28. Liu, R., Li, Y. & Wang, Q. Variations in water and CO_2_ fluxes over a saline desert in western China. *Hydrol. Process.* **26**, 513-522 (2012).

29. Liu, R. *et al.* High efficiency in water use and carbon gain in a wet year for a desert halophyte community. *Agr. Forest. Meteorol.* **162**, 127-135 (2012).

30. Du, Q. & Liu, H. Seven years of carbon dioxide exchange over a degraded grassland and a cropland with maize ecosystems in a semiarid area of China. *Agric., Ecosyst. Environ.* **173**, 1-12 (2013).

31. Liu, H. & Feng, J. Seasonal and interannual variations of evapotranspiration and energy exchange over different land surfaces in a semiarid area of China. *J. Appl. Meteorol. Clim.* **51**, 1875-1888 (2012).

32. Lei, H. & Yang, D. Interannual and seasonal variability in evapotranspiration and energy partitioning over an irrigated cropland in the North China Plain. *Agr. Forest. Meteorol.* **150**, 581-589 (2010).

33. Lei, H. M. & Yang, D. W. Seasonal and interannual variations in carbon dioxide exchange over a cropland in the North China Plain. *Global. Change. Biol.* **16**, 2944-2957 (2010).

34. Xiao, J. *et al.* Carbon fluxes, evapotranspiration, and water use efficiency of terrestrial ecosystems in China. *Agr. Forest. Meteorol.* **182-183**, 76-90 (2013).

35. Zhou, L., Zhou, G. & Jia, Q. Annual cycle of CO_2_ exchange over a reed (*Phragmites australis*) wetland in Northeast China. *Aquat. Bot.* **91**, 91-98 (2009).

36. Zhou, L., Zhou, G., Liu, S. & Sui, X. Seasonal contribution and interannual variation of evapotranspiration over a reed marsh (*Phragmites australis*) in Northeast China from 3-year eddy covariance data. *Hydrol. Process.* **24**, 1039-1047 (2010).

1. ^*^Corresponding to: juweimin@nju.edu.cn [↑](#footnote-ref-2)
